# The arginase 1/ornithine decarboxylase pathway suppresses HDAC3 to ameliorate the myeloid cell inflammatory response: implications for retinal ischemic injury

**DOI:** 10.1038/s41419-023-06147-7

**Published:** 2023-09-21

**Authors:** Esraa Shosha, Rami A. Shahror, Carol A. Morris, Zhimin Xu, Rudolf Lucas, Meghan E. McGee-Lawrence, Nancy J. Rusch, Ruth B. Caldwell, Abdelrahman Y. Fouda

**Affiliations:** 1 Department of Pharmacology and Toxicology, College of Medicine, University of Arkansas for Medical Sciences, Little Rock, AR USA; 2 Department of Clinical Pharmacy, Faculty of Pharmacy, Cairo University, Cairo, Egypt; 3 Vascular Biology Center, Augusta University, Augusta, GA USA; 4 Culver Vision Discovery Institute, Augusta University, Augusta, GA USA; 5 Department of Cellular Biology and Anatomy, Augusta University, Augusta, GA USA

**Keywords:** Microglia, Interleukins

## Abstract

The enzyme arginase 1 (A1) hydrolyzes the amino acid arginine to form L-ornithine and urea. Ornithine is further converted to polyamines by the ornithine decarboxylase (ODC) enzyme. We previously reported that deletion of myeloid A1 in mice exacerbates retinal damage after ischemia/reperfusion (IR) injury. Furthermore, treatment with A1 protects against retinal IR injury in wild-type mice. PEG-A1 also mitigates the exaggerated inflammatory response of A1 knockout (KO) macrophages in vitro. Here, we sought to identify the anti-inflammatory pathway that confers macrophage A1-mediated protection against retinal IR injury. Acute elevation of intraocular pressure was used to induce retinal IR injury in mice. A multiplex cytokine assay revealed a marked increase in the inflammatory cytokines interleukin 1β (IL-1β) and tumor necrosis factor α (TNF-α) in the retina at day 5 after IR injury. In vitro, blocking the A1/ODC pathway augmented IL-1β and TNF-α production in stimulated macrophages. Furthermore, A1 treatment attenuated the stimulated macrophage metabolic switch to a pro-inflammatory glycolytic phenotype, whereas A1 deletion had the opposite effect. Screening for histone deacetylases (HDACs) which play a role in macrophage inflammatory response showed that A1 deletion or ODC inhibition increased the expression of HDAC3. We further showed the involvement of HDAC3 in the upregulation of TNF-α but not IL-1β in stimulated macrophages deficient in the A1/ODC pathway. Investigating HDAC3 KO macrophages showed a reduced inflammatory response and a less glycolytic phenotype upon stimulation. In vivo, HDAC3 co-localized with microglia/macrophages at day 2 after IR in WT retinas and was further increased in A1-deficient retinas. Collectively, our data provide initial evidence that A1 exerts its anti-inflammatory effect in macrophages via ODC-mediated suppression of HDAC3 and IL-1β. Collectively we propose that interventions that augment the A1/ODC pathway and inhibit HDAC3 may confer therapeutic benefits for the treatment of retinal ischemic diseases.

## Introduction

Ischemic retinopathies including diabetic retinopathy, glaucoma, retinopathy of prematurity, and retinal artery or vein occlusion are leading causes of vision impairment. The pathophysiology of ischemic retinopathies involves a complex interplay of inflammation and oxidative stress [1]. We demonstrated earlier that one of the key protective mechanisms after retinal IR injury is the activation of the arginase enzyme (A1) [2–4]. Arginase converts the amino acid, L-arginine, to urea and L-ornithine. The enzyme has two isoforms, arginase 1 (A1) that is mainly cytosolic, and arginase 2 (A2) that is located primarily in mitochondria. Our recent reports using isoform-specific A1 and A2 global knockout mice indicate that A1 is protective against retinal ischemia whereas A2 exacerbates the disease [2, 5]. Interestingly the protective effect of A1 appears to rely on its expression in myeloid cells, because myeloid-specific deletion of A1 exacerbates acute retinal ischemia-reperfusion (IR injury. Conversely, intravitreal or systemic treatment with the investigational drug pegylated arginase 1 (PEG-A1) limits IR injury [2–4], suggesting that the A1 isoform could be leveraged for possible therapeutic advantage.

Retinal IR injury involves the activation and proliferation of microglia and resident macrophages, as well as recruitment of blood-borne monocytes [6]. These activated myeloid cells play either a protective or a deleterious role depending on their molecular profile and activation state. In contrast to macrophage classical activation that defends against pathogens, macrophage alternative activation is widely documented to play a reparative role in CNS diseases [7], and the A1 enzyme is a hallmark of alternatively activated macrophages (M2) [8]. Indeed, our studies in myeloid-specific A1 KO mice suggest a neuroprotective and anti-inflammatory role of myeloid A1 in retinal IR injury [2]. However, the mechanisms by which A1 in myeloid cells protects the retina from ischemic injury are poorly understood. A1 competes with the enzyme, inducible nitric oxide synthase (iNOS), for arginine as a shared substrate, thereby reducing nitric oxide (NO) production by activated macrophages. A1 converts arginine to L-ornithine, which subsequently is converted to polyamines by the ornithine decarboxylase (ODC) enzyme [9]. The polyamines play diverse physiological roles including participation in inflammation, tissue repair, and wound healing [9].

Microglia/infiltrating macrophages are the main sources of the inflammatory cytokines interleukin 1 beta (IL-1β) and tumor necrosis alpha (TNF-α), which are implicated in retinal IR injury [10–12]. The IL-1β gene is induced after retinal IR and remains upregulated for as long as 7 days [13]. It was shown to play a central role in retinal IR pathology [14]. Similarly, TNF-α is upregulated acutely after retinal IR and leads to cell necroptosis [15, 16]. Immunohistochemical analyses have confirmed that IL-1β and TNF-α are primarily produced by microglia/macrophages in the ischemic model of oxygen-induced retinopathy (OIR) [11, 17].

The histone deacetylases (HDACs) also have been implicated in the macrophage inflammatory response. The HDAC enzymes remove acetyl groups from the lysine amino acid in histone and non-histone proteins. Class I HDACs comprised of HDACs 1, 2, 3 and 8 regulate inflammatory gene expression [18]. Specifically, HDAC3 plays a central contributive role in the macrophage inflammatory response [19, 20]. It regulates inflammatory gene expression in lipopolysaccharide (LPS)-stimulated macrophages [20]. It also acts as a brake to repress expression of the A1 gene during macrophage alternative activation [19]. However, whether endogenous A1 or its therapeutic PEG-A1 analog influence HDAC3 expression and inflammatory functions has not been examined to our knowledge.

The goal of the present study was to identify the mechanisms responsible for the protective and anti-inflammatory influence of myeloid A1 in retinal IR injury. We provide evidence that A1 inhibits IL-1β and TNF-α expression in macrophages via activation of the ODC pathway. Furthermore, we report that A1/ODC suppresses TNF-α and macrophage metabolic switch to a pro-inflammatory glycolytic phenotype via inhibition of HDAC3 expression. Our findings highlight the A1-ODC signaling pathway as a critical protector from retinal IR injury and suggest that increasing A1 abundance coupled to HDAC3 inhibition may be a strong strategy to attenuate retinal IR injury.

## Materials and methods

### Mouse colony and retinal ischemia-reperfusion (IR) injury model

All experiments were performed in accordance with the ARVO Statement for the Use of Animals in Ophthalmic and Vision Research and were approved by the Institutional Animal Care and Use Committee. Male wild-type (WT) C57BL/6J and global heterozygous A1^+/-^ KO mice (10 to 12-week-old) were anesthetized using a ketamine/xylazine mixture given intraperitoneally and then subjected to retinal ischemia followed by reperfusion to achieve ischemia-reperfusion (IR) injury as described earlier [5]. Briefly, a 30-gauge needle connected to a raised saline bag was inserted into the anterior chamber of the right eye to raise the intraocular pressure and induce ischemia for 60 min followed by needle removal to allow reperfusion. The left eye served as a sham control. Mice were deeply anesthetized and sacrificed by cervical dislocation at indicated time points.

A1^+/-^ KO mice originally obtained from Dr. Stephen D Cederbaum were bred to WT and A1^+/-^ and WT littermates were used for experiments. Within the same cage of KO and controls, mice were randomly selected for IR injury without looking at the ear tag. There was no blinding since we did not assess phenotypic outcomes.

### Generation of myeloid-specific KO mice

Myeloid-specific KO mice were generated, and male myeloid KO and floxed littermates were used to obtain primary macrophages lacking the gene of interest:

Myeloid-specific A1 and A2 KO mice: C57BL/6J A1 and A2 floxed mice were crossed with mice expressing the myeloid lineage-specific promoter of the lysozyme 2 gene (Lyz2) (LysM cre, Stock No. 004781) as we previously described [2, 21]. Both mouse models were characterized earlier to confirm myeloid-specific deletion [2, 21].

Myeloid-specific HDAC3 KO mice: C57BL/6J floxed mice with LoxP sites on either side of exons 7 of HDAC3 (*HDAC3*^*f/f*^, originally developed by Dr. Scott W. Hiebert [22]) were kindly provided by Dr. McGee-Lawrence (Augusta University) and bred in our colony. These mice were crossed with LysM cre mice (Stock No. 004781) to generate HDAC3 myeloid-specific KO mice.

### Fluorescent immunolabeling

Eyeballs were fixed in 4% paraformaldehyde (PFA) and then dissected into retina flat mounts or cryoprotected in 30% sucrose followed by embedding in optimal cutting temperature (O.C.T.) and cryosectioning. Retina flatmounts or cryostat sections were permeabilized in 0.1 % triton X-100 and then blocked by the addition of 10% donkey serum and 1% bovine serum albumin (BSA) for 1 h. Primary antibodies (1:200) including IL-1β (R&D Systems, Cat. # AF-401-NA), Iba-1 (FUJIFILM Wako, Cat. # 019-19741), and HDAC 3 (BD Biosciences, Cat. # 611124) were incubated overnight at 4 °C followed by washing in PBS and incubation of fluorescent secondary antibodies (1:500) at room temperature for 1 h as previously described [5].

### Primary macrophage isolation and culture

Peritoneal macrophages: Thioglycolate-elicited peritoneal macrophages were isolated from male mice peritoneal lavage as described earlier [2]. Cells were plated in 12-well tissue culture plates and incubated overnight with LPS (100 ng/mL) with or without PEG-A1 (1 µg/ml), DFMO (5 mM), ornithine (5 mM), and RGFP966 (2 µM or 10 µM). Cells were collected the next morning after 18 h of incubation.

Bone marrow-derived macrophages (BMDMs): cultures were prepared from male mice as previously described [2]. Briefly, femurs and tibias were harvested and flushed with 20−25 ml sterile PBS. Cells in PBS were spun down and re-suspended in differentiation medium (DMEM high glucose containing 20% FBS, 20% L929 conditioned media, and 1% P/S) then subsequently plated on uncoated 100 mm dishes. Media was changed on day 4 and on day 7, cells were rinsed with sterile PBS and gently scraped, collected, and re-suspended in normal growth media (DMEM high glucose containing 20% FBS, and 1% P/S), and then plated for experiments. After the differentiation of bone marrow cells for 7 days, BMDMs were plated into Seahorse 96-well plates for Seahorse experiments described below.

### Seahorse XF96 Mito stress test and glycolysis stress test

Mitochondrial function and glycolysis machinery were evaluated as previously described using the Mito stress test (Agilent, Catalog # 103015-100, Santa Clara, CA) and the Glycolysis stress test (Agilent, catalog # 103020-100), respectively [21]. The Seahorse XF96e instrument was used to measure oxygen consumption rate (OCR) and extracellular acidification rate (ECAR) as indicators of mitochondrial respiration and glycolysis, respectively. BMDMs were isolated as described above, then seeded into Seahorse cell culture plates at a cell density of 40k/well. Corner wells (A1, A12, H1, and H12) were used as background wells. Plates were left under the hood for 1 h to ensure even distribution of cells, then cells were checked under the microscope and put in the incubator to grow. After 7 h, cells were treated with or without LPS (100 ng/ml) and RGFP966 (2 µM) or DMSO as a solvent control overnight. The day before the assay, the Seahorse media for the Mito stress test was prepared according to manufacturer’s instructions and supplemented with 4 mM glutamine (Gemini Bio Products, West Sacramento, CA), 25 mM glucose (Sigma) and 1 mM sodium pyruvate (Agilent). The media for the Glycolysis stress test was supplemented with 4 mM glutamine. Both assays were conducted according to the manufacturer’s instructions. The final concentrations of injected compounds used in these studies were: oligomycin (1 µM), carbonyl cyanide-p-trifluoromethoxyphenyl-hydrazon (FCCP, 1 µM), and rotenone/antimycin A (0.5 µM). At the end of the assays, the cells were collected in RIPA buffer for protein estimation. The data were collected and analyzed using Wave software (Agilent).

### ATP rate assay

The Agilent Seahorse XF Real-Time ATP Rate Assay was conducted following the manufacturer’s recommendations using the ATP rate assay kit (Agilent, Catalog # 103592-100). BMDMs were isolated and plated, then treated with LPS as described above. The Seahorse media for the ATP rate assay was prepared according to the manufacturer’s instructions the day before the assay and supplemented with 15 mM glucose (Sigma), 4 mM glutamine (Gemini) and 1 mM sodium pyruvate (Agilent). The assay was conducted according to the manufacturer’s protocol. The final concentrations of the injection compounds used in this assay were oligomycin (1 µM) and rotenone/antimycin A (0.5 µM). At the end of the assays, the cells were collected in RIPA buffer for protein estimation. The data were collected and analyzed using Wave software (Agilent).

### Western blotting

Retinas were collected, snap-frozen, and stored at −80 ^o^C until analysis. Frozen retinas or cells were homogenized in RIPA buffer (Thermofisher Scientific). Western blotting was conducted as previously described [2]. The primary antibodies used were IL-1β (R&D Systems, Cat. # AF-401-NA), Iba-1 (FUJIFILM Wako, Cat. # 019-19741), p-NF-κB (Cell Signaling, Cat. # 3033), T-NF-κB (Cell Signaling, Cat. # 4764), TNF-α (Abcam, Cat. # ab1793), HDAC1 (Cell signaling, Cat. # 5356), HDAC2 (Cell Signaling, Cat. # 5113), HDAC3 (BD Biosciences, Cat. # 611124), arginase 1 (GeneTex; Cat. # GTX109242), arginase 2 (Santa Cruz Biotechnology, Cat. # Sc-20151), tubulin (Sigma, Cat. # T9026), and β-actin (Sigma, Cat. # A1978-200UL). Secondary antibodies (GE Healthcare) were diluted 1:2000 in 5% milk.

### Quantitative reverse transcriptase PCR (RT-PCR)

Total RNA was extracted from macrophages using TRIzol reagent (Invitrogen) and converted to cDNA using M-MLV reverse transcriptase (Invitrogen). Quantitative PCR was performed as previously described and data were normalized to HPRT [2]. Fold-change between levels of different transcripts was calculated by the ΔΔCT method. Forward and reverse primers were as follows:

HDAC1 (f: 5′-TGCTCGCTGCTGGACTTAC-3′, r: 5′-GTAGGGCAGCTCATTAGGGATCT-3′);

HDAC2 (f: 5′-GCGTACAGTCAAGGAGGCGGC-3′, r: 5′-GGCTTCATGGGATGACCCTGGC-3′);

HDAC3 (f: 5′-CACCAAGAGCCTTGATGCCTT-3′, r: 5′-GCAGCTCCAGGATACCAATTACT-3′);

HDAC8 (f: 5′-TGCGACGGAAATTTGACCG-3′, r: 5′-TTGTGCAGGGACACAGTCAT-3′);

HDAC10 (f: 5′-TGGACCTTGCAGATGATGGGA-3′, r: 5′-GGCTCAGAAACCCTCCAGTT-3′);

Hprt (f: 5′-TCAGTCAACGGGGGACATAAA-3′, r:GGGGCTGTACTGCTTAACCAG)

Gene expression was performed using an ABI StepOne Plus Thermocycler (Applied Biosystems) and SYBR Green dye.

### Nuclear factor kappa B (NF-κB) translocation assay

NF-κB nuclear translocation was examined using fluorescence imaging as previously described [23]. BMDMs were grown on 35 mm Petri dishes with glass bottoms (Corning Inc.). Cells were stimulated with LPS ± PEG-A1 for 1 h, then washed and fixed in 4% PFA for 15 min. The fixed cells were stored at 4 °C in PBS. Immunocytochemistry was conducted by blocking in PBS containing 1% BSA and 10% donkey serum for 30 min at room temperature, then incubating overnight at 4 °C with anti-p65 primary antibody (1:100, Cell Signaling, Cat. # 4764). Subsequently, cells were washed, and a secondary antibody (donkey anti-rabbit Alexa-Fluor 488, Invitrogen) was applied for 1 h at room temperature before counterstaining with Vectashield (Vector Laboratories) containing DAPI (Vector labs) to allow visualization of nuclei. Z-stack images in the nuclei plane were taken using a Zeiss 780 inverted confocal microscope (Carl Zeiss). DAPI images were subtracted from the NF-κB images using the image calculator feature in ImageJ software to create images with only cytoplasmic NF-κB signal. Fluorescent signal was quantified on total and cytoplasmic NF-κB images and nuclear NF-κB signal was obtained by subtraction. Results were presented as the ratio of nuclear to cytoplasmic intensities.

### Multiplex analysis of cytokines in retina tissue

Cytokine concentrations were quantified with the multiplex MCYTOMAG-70K assay (EMD Millipore, Sigma-Aldrich), according to the manufacturer’s instructions.

### Statistical analysis

Statistics and graphs were performed using GraphPad Prism 9 software and data were presented as mean ± standard error (SE). A *p*-value < 0.05 was considered statistically significant. The difference between two groups was determined by student’s *t*-test while comparisons between multiple groups were analyzed by analysis of variance (ANOVA) with Tukey’s post hoc test. The sample size was determined based on previous lab results.

## Results

### IL-1β and TNF-α are markedly upregulated after retinal ischemic injury

**Fig. 1:**
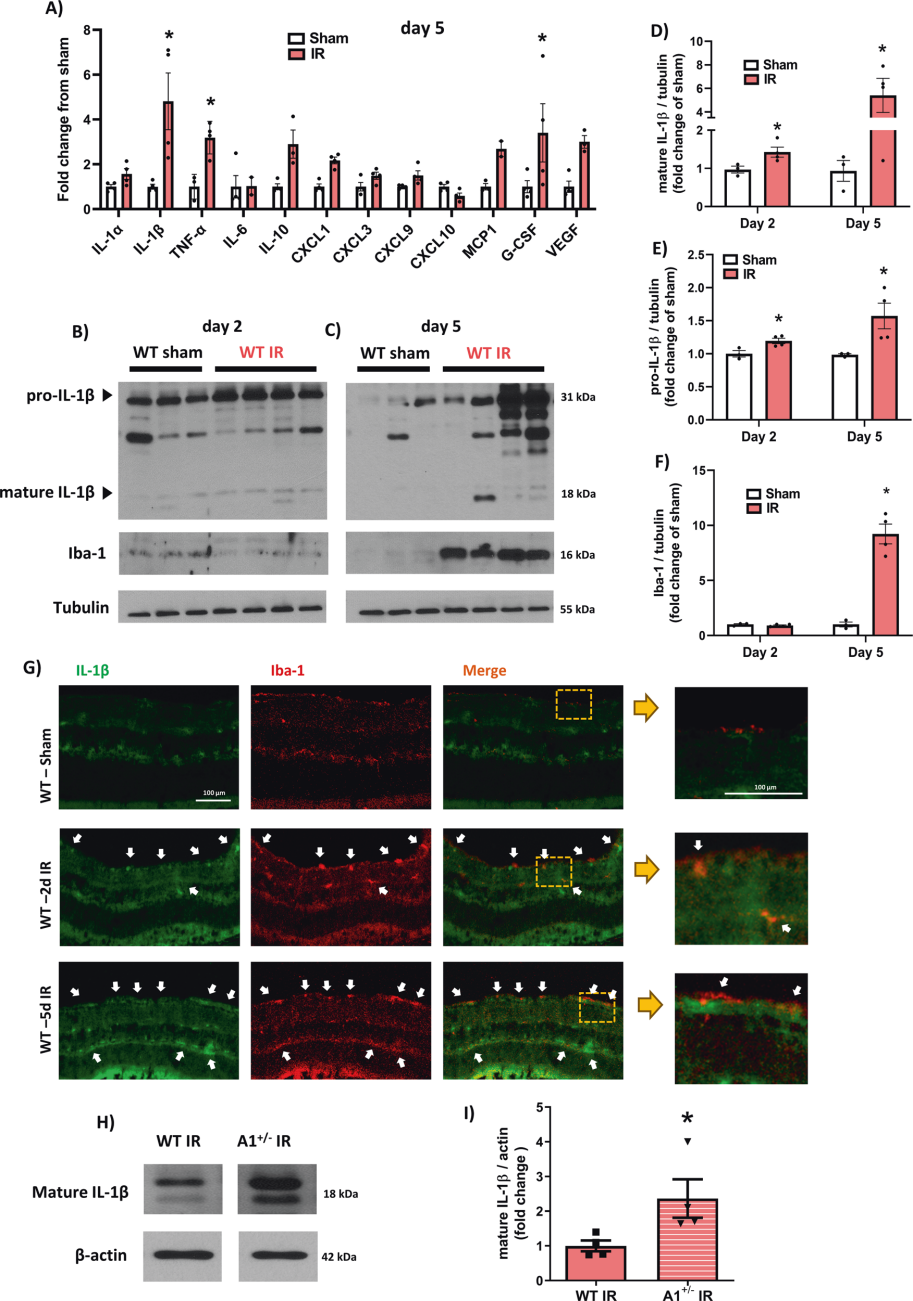
Induction of cytokines including TNF-α and IL-1β after retinal IR injury and localization of IL-1β in macrophage/microglia cells. **A** Multiplex array shows strong upregulation of IL-1β, TNF-α, and G-CSF in retinal lysates at day 5 after IR injury. *N* = 3–4 per group. *p < 0.05 vs. sham. **B**–**F** Western blotting on retina lysates at days 2 and 5 after IR injury and quantification show significant upregulation of pro- and mature forms of IL-1β with a higher increase at day 5. The macrophage/microglia cell marker Iba-1 showed a strong upregulation at day 5 but not day 2 after IR injury. Tubulin was used as a loading control. *N* = 3–4 per group. *p < 0.05 vs. respective sham. **G** Immunolabeling on retina cross-sections shows co-localization (indicated by white arrows and magnified insets of the yellow boxes) of IL-1β with Iba-1 positive microglia/macrophages at days 2 and 5 after IR injury. *N* = 3 per group. *p < 0.05 vs. respective sham. **H**, **I** Expression level of mature IL-1β is significantly higher in retinas from hemizygous A1 KO mice (A1^+/-^) at day 5 after IR injury compared to WT mice as measured by western blotting. Actin was used as a loading control. *N* = 4 per group. *p < 0.05 vs. WT IR.

The retinal ischemia-reperfusion (IR) injury model induced by rising intraocular pressure is associated with an acute inflammatory response and activation of microglia and macrophages. Microglial proliferation and macrophage infiltration reach their peak at 5 days post-injury [24]. We conducted a multiplex array on WT retinas on day 5 after IR to determine the predominant myeloid-associated cytokine/chemokine molecules induced in this model that may contribute to the inflammatory response (Fig. 1A). IL-1β and TNF-α which are associated with macrophages/microglia activation were significantly upregulated after retinal IR injury. The pro-inflammatory cytokine IL-1β exhibited the greatest increase, five-fold, whereas TNF-α increased three-fold. Granulocyte colony-stimulating factor (G-CSF) similarly increased three-fold after retinal IR but this protective cytokine is primarily located in retinal neurons and not myeloid cells [25, 26].

We reported earlier that TNF-α is increased acutely in retinal IR injury, and that A1 deletion enhances TNF-α expression in the injured retina, indicating that endogenous A1 suppresses this inflammatory response [2]. Additionally, PEG-A1 treatment of isolated macrophages attenuates expression of TNF-α, pro-IL-1β, and iNOS, implicating these proteins as potential downstream signaling pathways by which A1 confers anti-inflammatory protection. Here, we show an increased expression of the immature form of IL-1β (pro-IL-1β) and the mature active form (IL-1β) by Western blot analysis at 5 days after retinal IR injury in our model (Fig. 1C–E), thereby validating the IL-1β multiplex results (Fig. 1A); IL-1β was increased less at day 2 compared to day 5 as evidence of a temporally progressing event (Fig. 1B–E). The increase in IL-1β protein levels at day 5 after retinal IR coincided with a nine-fold increase in ionized calcium-binding adaptor protein-1 (Iba-1), a shared marker unique to microglia and macrophages (Fig. 1C, F). Flatmount staining showed Iba-1 positive cells exhibited a more ameboid morphology with enlarged soma and retracted processes at day 2 after injury, with a strong increase in number of these cells and change in morphology to a rod-like phenotype at day 5 (Fig. S1) [27]. Furthermore, immunolocalization studies at days 2 and 5 after IR showed IL-1β to be strongly expressed within or in close proximity to Iba-1 positive microglia/macrophages (Fig. 1G). To determine whether A1 regulates IL-1β expression in retina after IR injury, we compared IL-1β expression between retinas from WT and A1 heterozygous KO mice (A1^+/-^) at 5 days after IR. The expression of IL-1β was increased in A1^+/-^ retinas as compared with WT retinas after IR, implying that endogenous A1 suppresses the expression of IL-1β after IR (Fig. 1H, I), similar to its known suppression of TNF-α in the same model [2]. Collectively, our current and earlier findings indicate that retinal IR injury is associated with the induction of IL-1β and TNF-α and the production of both inflammatory cytokines can be suppressed by A1.

### Arginase 1 but not Arginase 2 signals through ornithine to suppress IL-1β and TNF-α expression in LPS-stimulated macrophages

**Fig. 2:**
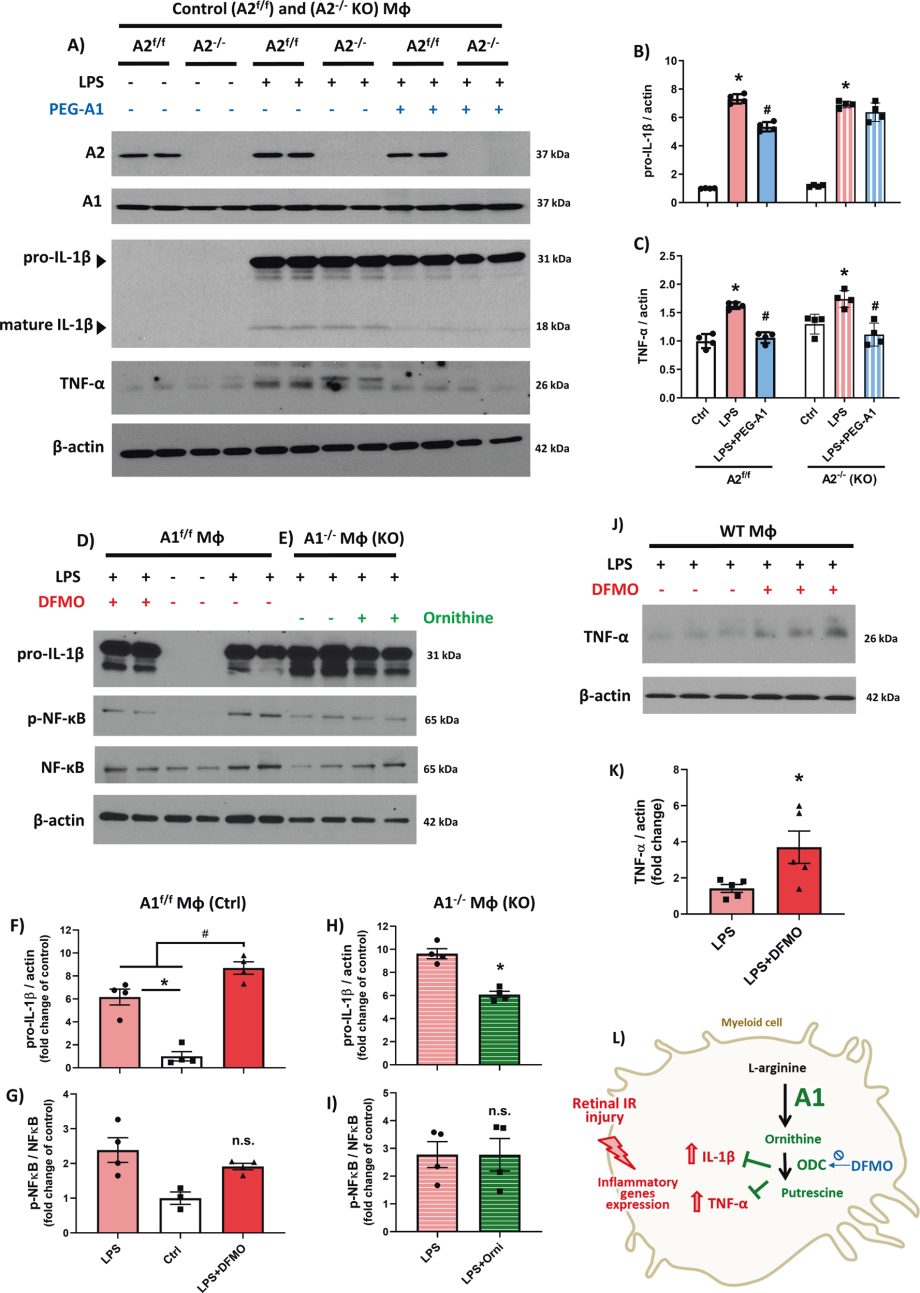
Arginase 1 but not Arginase 2 signals through ornithine to suppress IL-1β and TNF-α expression in LPS-stimulated macrophages. **A**–**C** Deletion of arginase 2 in macrophages from A2^-/-^ mice did not alter LPS-induced elevations of pro-IL-1β and TNF-α; macrophages from A2^f/f^ mice served as control. In contrast, treatment of A2^-/-^ or A2^f/f^ macrophages with PEG-A1 (1 µg/ml) reduced expression of both cytokines. *N* = 4 per group. *p < 0.05 vs. respective vehicle group, ^#^*p* < 0.05 vs. respective LPS group. **D**, **F** Western blotting and quantification showed increased IL-1β expression after ODC inhibition with DFMO (5 mM) in LPS-stimulated macrophages. *N* = 4 per group. *p < 0.05 vs. LPS groups, ^#^*p* < 0.05 vs. control and LPS groups. **E**, **H** Supplementation with ornithine (5 mM) reduced LPS-induced IL-1β expression in A1^-/-^ macrophages. *N* = 4 per group. *p < 0.05 vs. LPS. **G**, **I** NF-κB phosphorylation was unchanged after exposure of A1^f/f^ and of A1^-/-^ macrophages to either DFMO or ornithine (5 mM). *N* = 3–4 per group. n.s. = not statistically significant. **J**, **K** Inhibition of ODC activity by DFMO (5 mM) increased TNF-α expression in LPS-stimulated macrophages from WT mice. *N* = 3–5 per group. *p < 0.05 vs. control and LPS groups. **L** Schematic of Fig. 2 experiments showing A1/ODC pathway suppresses IL-1β and TNF-α expression in stimulated macrophages.

An increased availability of A1 in activated macrophages has been observed to dampen induction of IL-1β, TNF-α, and iNOS as components of its broad anti-inflammatory effect, although it’s unclear whether parallel changes in A2 may confer confounding effects. Here, we show that upregulation of IL-1β and TNF-α in LPS-activated macrophages (100 ng/mL of LPS) was unaltered in macrophages isolated from A2 knockout (A2^-/-^) mice, whereas PEG-A1 (1 µg/ml) treatment suppresses IL-1β and TNF-α expression in the activated macrophages (Fig. 2A–C) suggesting an exclusive effect of A1 on expression of these two cytokines. Importantly, deletion of A2 in A2^-/-^ mice did not affect A1 expression in the retina (Fig. 2A and S2A). Next, we used LPS-activated macrophages to explore whether A1 suppresses IL-1β and TNF-α by signaling through the polyamine pathway. Using this pathway, A1 converts arginine to L-ornithine, which subsequently is converted to polyamines by ornithine decarboxylase (ODC). Inhibition of ODC by difluoromethylornithine (DFMO, 5 mM) increased pro-IL-1β (Fig. 2D, F) and TNF-α (Fig. 2J, K) expression in response to LPS stimulation, suggesting that ODC-mediated formation of polyamines normally limits induction of IL-β1 and TNF-α during macrophage activation [2]. Of note, DFMO did not alter A1 protein expression in WT macrophages (Fig. S2B, C). Furthermore, ornithine supplementation (5 mM) reduced pro-IL-1β expression in A1^-/-^ macrophages (Figs. 2E, H and S2D), providing evidence that increasing the substrate for ODC with a presumed increase in downstream polyamines negatively regulates IL-1β expression [2]. Collectively, our results implicate ODC–derived polyamine signaling in the suppression by A1 of IL-1β and TNF-α in activated macrophages.

Since IL-1β and TNF-α are primary response genes of the transcription factor NF-κB canonical pathway [28], we considered the possibility that the A1/ODC/polyamine pathway acts through NF-κB to suppress cytokine production. However, neither ODC inhibition by DFMO nor ornithine supplementation altered the expression of phosphorylated NF-κB in either A1^f/f^ or A1^-/-^ macrophages activated by LPS (Fig. 2D, E, G, I). We also employed immunocytochemistry to examine whether increasing A1 availability would enhance the nuclear translocation of NF-κB. However, NF-κB translocation was unchanged in macrophages treated with PEG-A1 (Fig. S2E, F) or in A1^-/-^ macrophages as compared to A1^f/f^ controls (Fig. S2G, H). Therefore, we concluded that the A1/ODC/polyamine pathway negatively regulates IL-1β and TNF-α expression independently of NF-κB.

### The A1/ODC pathway inhibits HDAC3 expression in LPS-stimulated macrophages

**Fig. 3:**
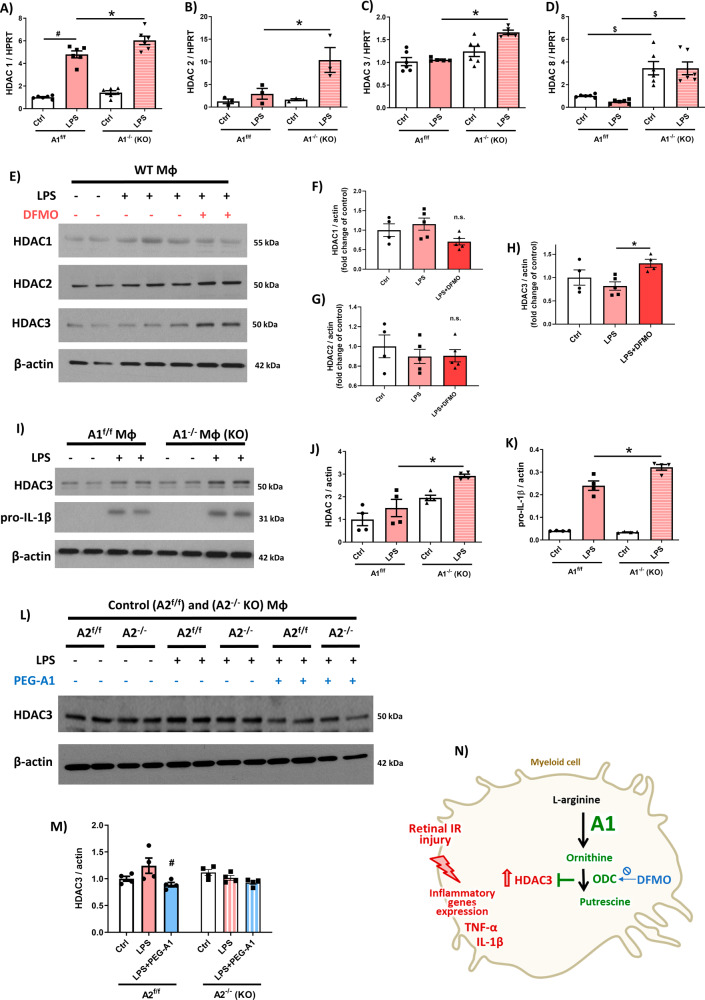
The A1/ODC pathway inhibits HDAC3 expression in macrophages. **A**–**D** RT-PCR reveals significant upregulation of HDAC1, 2, and 3 but not HDAC8 in A1^-/-^ macrophages after LPS treatment. *N* = 3–6 per group, *p < 0.05 vs. A1^f/f^ LPS group, ^#^*p* < 0.05 vs. A1^f/f^ ctrl group, ^$^*p* < 0.05 vs. respective A1^f/f^ groups. **E**–**H** Western blotting showed significant upregulation of HDAC3 but not HDAC2 or HDAC1 in LPS-activated macrophages exposed to DFMO (5 mM) to inhibit the ODC enzyme. *N* = 4–5 per group. *p < 0.05 vs. LPS group, n.s. = not statistically significant. **I**–**K** A1^-/-^ macrophages showed enhanced upregulation of HDAC3 and IL-1β in response to LPS activation compared to A1^f/f^ macrophages. *N* = 4 per group, *p < 0.05 vs. A1^f/f^ LPS group. **L**, **M** A2 KO macrophages showed no change in HDAC3 expression as compared to floxed control while PEG-A1 treatment reduced it. *N* = 4, ^#^*p* < 0.05 vs. A2^f/f^ LPS group. **N** Schematic of Fig. 3 experiments showing A1/ODC pathway inhibits HDAC3 expression in stimulated macrophages.

Our next set of studies investigated the downstream event(s) by which A1 signals through ODC-derived polyamines to suppress IL-1β and TNF-α expression in activated macrophages as a possible mechanism of protection from retinal IR injury. Because polyamines are known to modulate gene expression by altering histone acetylation [29, 30], we examined whether A1 regulates macrophage inflammatory gene expression by epigenetic modification. We initially explored whether A1 deletion in A1^-/-^ macrophages regulates the mRNA levels of the Class 1 HDACs (HDAC1, 2, 3, 8), which have been implicated in the macrophage inflammatory response. The basal mRNA levels of HDAC1, 2, 3 but not 8 were not significantly different between A1^f/f^ and A1^-/-^ macrophages (Fig. 3A–D). Activation of A1^f/f^ macrophages by LPS only increased HDAC1 transcript. However, LPS activation of A1^-/-^ macrophages resulted in a more pronounced induction of mRNAs coding for HDAC1, 2, and 3 implying that endogenous A1 serves to limit HDAC-mediated epigenetic repression in activated macrophages. Interestingly, there was no difference in ODC or the polyamine deacetylase, HDAC10, expression between control and A1^-/-^ macrophages even though both were upregulated with LPS (Fig. S3A, B).

Subsequent studies identified HDAC3 as the downstream target of A1/ODC/polyamine signaling. In these experiments, macrophages treated with DFMO to inhibit ODC showed increased expression of HDAC3 protein but not HDAC1 and HDAC2 (Fig. 3E–H). HDAC3 protein expression also was higher in LPS-activated A1^-/-^ macrophages compared to control A1^f/f^ (Fig. 3I, J), a finding associated with higher pro-IL-1β (Fig. 3I, K). The negative regulation by A1 of HDAC3 expression was confirmed by treating LPS-stimulated A1^-/-^ macrophages with PEG-A1. HDAC3 expression was dampened by PEG-A1 in activated A1^-/-^ macrophages (Fig. S3C). In contrast, A2 deletion in A2^-/-^ macrophages failed to affect HDAC3 expression with LPS stimulation (Fig. 3L, M). Reciprocally, we examined whether HDAC3 regulates A1 expression using HDAC3^-/-^ macrophages. As reported previously [19], HDAC3 deletion increased A1 expression suggesting bi-directional inhibition between A1 and HDAC3 (Fig. S3D, E).

### Involvement of HDAC3 in the augmented inflammatory response of macrophages deficient in the A1/ODC pathway

**Fig. 4:**
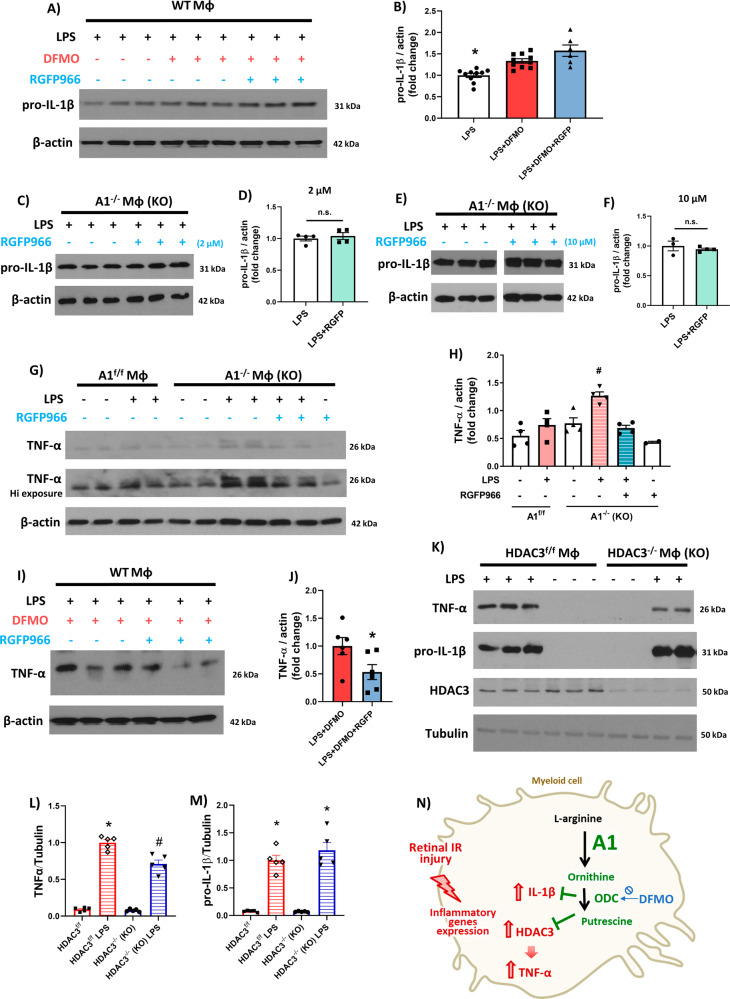
HDAC3 inhibition ameliorates expression of TNF-α but not IL-1β in macrophages deficient in the A1/ODC pathway. **A**, **B** Western blotting and quantification show significant upregulation of LPS-induced increases in pro-IL-1β with DFMO (5 mM) co-treatment while co-treatment with the HDAC3 inhibitor, RGFP966 (2 µM), had no effect. *N* = 6–10, *p < 0.05 vs. other groups. **C**–**F** HDAC3 inhibition with RGFP966 (2 or 10 µM) did not affect pro-IL-1β expression upon LPS stimulation in A1 KO macrophages. *N* = 3–4, n.s. = not statistically significant. **G**, **H** HDAC3 inhibition with RGFP966 (2 µM) dampened the LPS-induced increase in TNF-α expression in A1 KO macrophages. *N* = 4 ^#^p < 0.05 vs. all other groups. **I**, **J** HDAC3 inhibition with RGFP966 (2 µM) dampened TNF-α expression in WT macrophages treated with TNF and the ODC inhibitor, DFMO (5 mM). N = 6, *p < 0.05 vs. LPS + DFMO. **K**–**M** HDAC3 KO macrophages showed decreased expression of TNF-α but not pro-IL-1β upon LPS stimulation. *N* = 5, *p < 0.01 vs. HDAC3^f/f^ LPS. **N** Schematic of Fig. 4 experiments showing A1/ODC pathway suppresses TNF-α through inhibiting HDAC3 expression in stimulated macrophages.

We next examined the potential impact of A1/ODC-mediated reduction of HDAC3 expression on the macrophage inflammatory response by treating LPS-activated macrophages with the HDAC3 inhibitor, RGFP966 (2 µM). WT macrophages were first incubated with the ODC inhibitor, DFMO, to prevent any crosstalk between A1/ODC signaling and HDAC3. Contrary to our expectations, adding 2 μM RGFP966 to the LPS-activated macrophages did not lower IL-1β expression (Fig. 4A, B). Similar additions of 2 or 10 μM RGFP966 also did not affect IL-1β expression in activated A1^-/-^ macrophages (Fig. 4C–F). Interestingly, inhibition of HDAC3 by the addition of 2 μM RGFP966 ameliorated the increased expression of TNF-α expression in activated A1^-/-^ macrophages (Fig. 4G, H). Likewise, HDAC3 inhibition by 2 μM RGFP966 dampened TNF-α expression in DFMO-treated WT macrophages (Fig. 4I, J). Finally, we directly determined whether HDAC3 suppresses TNF-α but not IL-1β expression by isolating macrophages from HDAC3^-/-^ mice (Fig. 4K–M). Indeed, deletion of HDAC3 suppressed TNF-α but not IL-1β expression in LPS-activated HDAC3^-/-^ macrophages. Thus, HDAC3 appears to be a downstream regulator of the A1/ODC/polyamine signaling pathway that suppresses TNF-α but not IL-1β expression in activated macrophages.

### A1 dampens the LPS-induced macrophage metabolic switch to a glycolytic phenotype

**Fig. 5:**
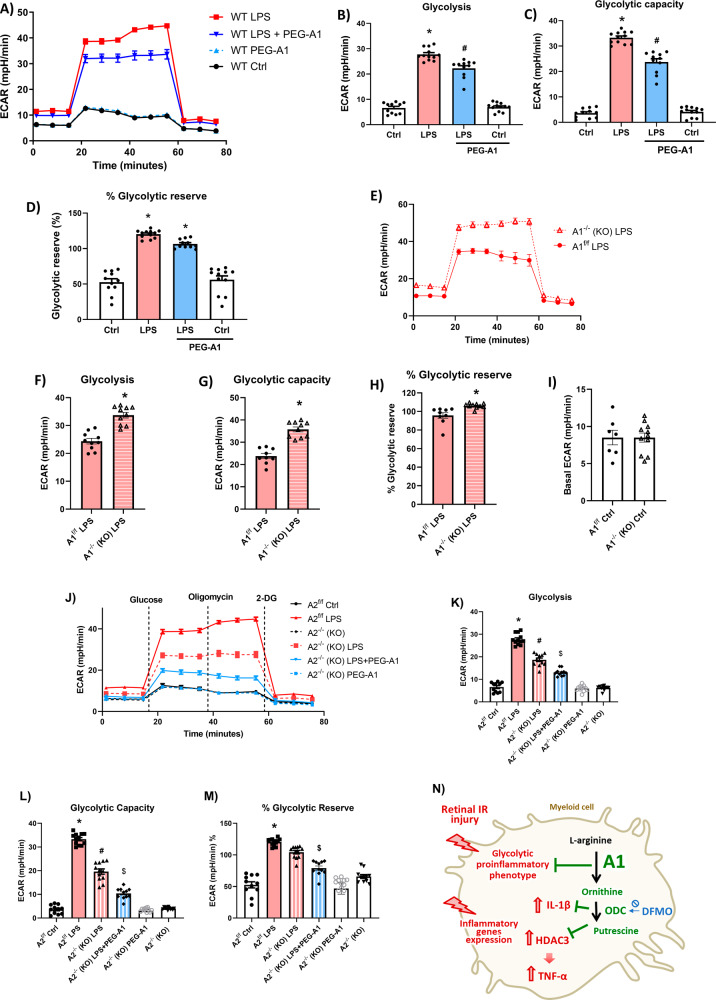
LPS-induced glycolysis in macrophages is dampened by PEG-A1 treatment and enhanced by A1 deletion. **A**–**D** Seahorse glycolysis stress test on BMDMs showed enhanced glycolytic response upon LPS stimulation while PEG-A1 treatment significantly reduced it as evidenced by decreased glycolysis, glycolytic capacity, and % glycolytic reserve. *N* = 11–12 *p < 0.05 vs. ctrl, ^#^*p* < 0.05 vs. LPS. **E**–**H** A1 KO macrophages showed an increased glycolytic response upon LPS stimulation as evident by increased glycolysis, glycolytic capacity, and % glycolytic reserve. *N* = 9–10, *p < 0.05 vs. A1^f/f^ LPS. **I** Basal ECAR in unstimulated macrophages was similar to floxed controls. *N* = 7–11. **J**–**M** Seahorse glycolysis stress test showed decreased glycolytic response in A2 KO macrophages stimulated with LPS while PEG-A1 co-treatment further decreased it which was evident in glycolysis, glycolytic capacity, and % glycolytic reserve. *N* = 11–12 *p < 0.05 vs. A2^f/f^ Ctrl, ^#^*p* < 0.05 vs. A2^f/f^ LPS, ^$^*p* < 0.05 vs. A2^-/-^ LPS. **N** Schematic of Fig. 5 experiments showing A1/ODC pathway inhibiting the glycolytic pro-inflammatory phenotype in stimulated macrophages.

It is well established that ‘M1-like’ macrophages stimulated with LPS undergo metabolic programming to become reliant mainly on glycolysis rather than mitochondrial respiration (also known as the Warburg effect) [31]. Furthermore, recent reports indicate a critical role of glycolytic flux in the macrophage inflammatory response [32–35]. Therefore, we conducted Seahorse metabolic assays to determine the impact of A1 treatment on the glycolytic response of macrophages to LPS stimulation. Assessment of glycolytic activity using extracellular acidification rate (ECAR) showed that PEG-A1 treatment dampened the LPS-induced glycolysis and glycolytic capacity in macrophages (Fig. 5A–D). Furthermore, A1^-/-^ macrophages exhibited increased glycolysis, glycolytic capacity and % glycolytic reserve in response to LPS stimulation compared to A1^f/f^ controls (Fig. 5E–H), while basal ECAR was not significantly different between control and A1 KO untreated macrophages (Fig. 5I). Investigation of the role of the other arginase isoform A2 on LPS-induced macrophage reprogramming revealed a significant decrease in glycolysis in LPS-stimulated macrophages lacking A2 (Fig. 5J–M). Interestingly, PEG-A1 treatment further decreased the glycolytic response of LPS-stimulated A2 KO macrophages (Fig. 5J–M). Collectively, the Seahorse analyses confirmed that A1 dampens the macrophage inflammatory glycolytic flux, an effect that is opposite from that of the A2 isoform.

### HDAC3 contributes to the enhanced glycolytic response of LPS-stimulated A1 KO macrophages

**Fig. 6:**
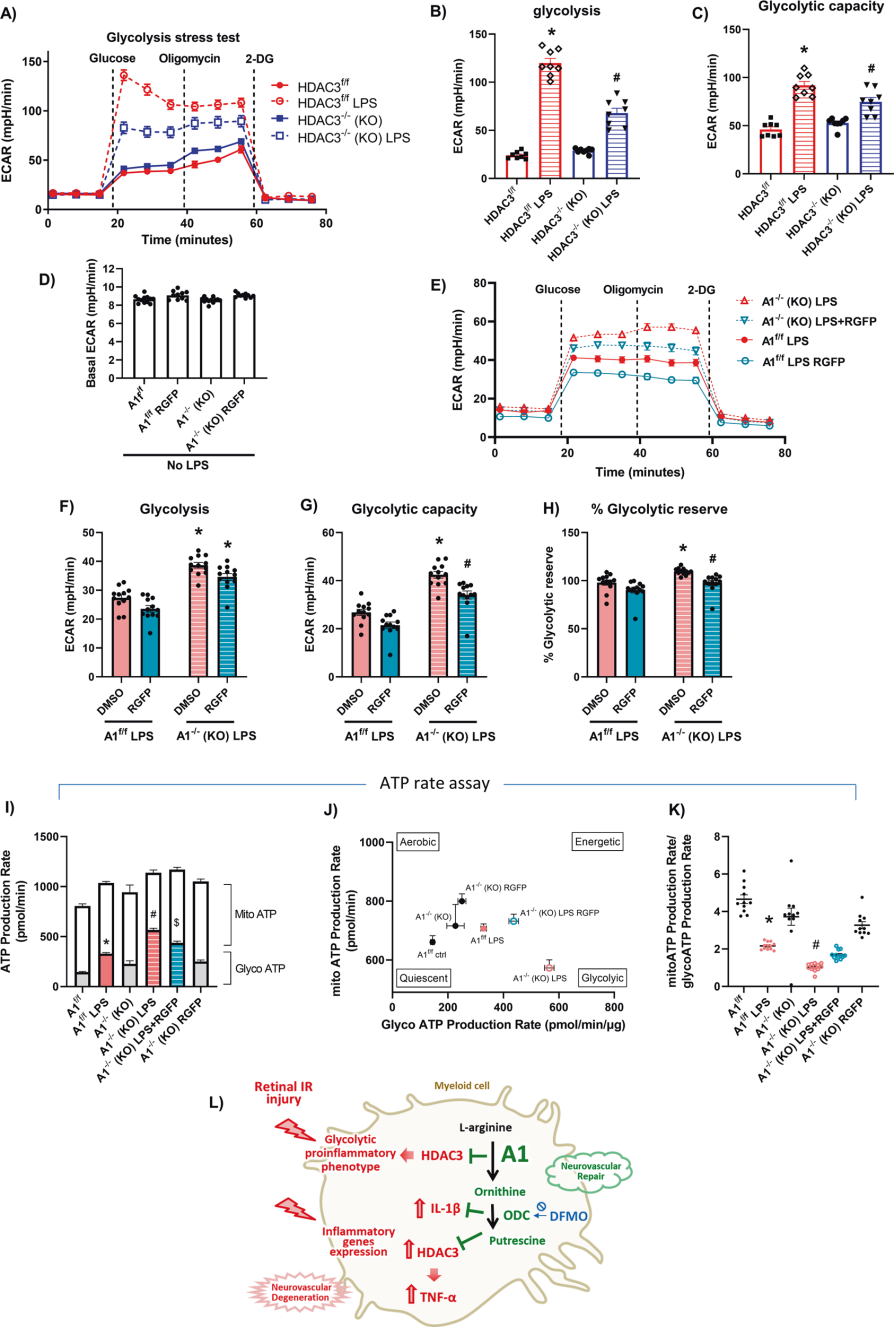
Involvement of HDAC3 in the augmented glycolytic response of stimulated A1 KO macrophages. **A**–**C** HDAC3 KO BMDMs showed decreased glycolytic response upon LPS stimulation as compared to floxed control which is evident by decreased glycolysis and glycolytic capacity. *N* = 7–8, *p < 0.05 vs. HDAC3^f/f^, ^#^*p* < 0.05 vs. HDAC3^f/f^ LPS. **D** Seahorse analysis on unstimulated macrophages shows no change in basal ECAR with A1 deletion or RGFP966 treatment. *N* = 10–12. **E**–**H** Seahorse glycolysis stress test showed decreased glycolytic capacity and % glycolytic reserve but not glycolysis in LPS-stimulated A1 KO macrophages co-treated with the HDAC3 inhibitor, RGFP966 (2 µM). *N* = 10–12 *p < 0.05 vs. respective A1^f/f^ LPS groups, ^#^*p* < 0.05 vs. DMSO A1^-/-^ LPS. **I** Seahorse ATP rate assay showed decreased glycolytic ATP production in LPS-stimulated A1 KO macrophages co-treated with the HDAC3 inhibitor, RGFP966 (2 µM). *N* = 11–12, *p < 0.05 vs. A1^f/f^, ^#^*p* < 0.05 vs. A1^f/f^ LPS, ^$^*p* < 0.05 vs. A1^-/-^ LPS. **J**, **K** Plotting mitochondrial ATP production against glycolytic ATP production and calculating mitochondrial ATP to glycolytic ATP ratio showed an enhanced glycolytic phenotype in LPS-stimulated A1 KO macrophages which was ameliorated with the HDAC3 inhibitor, RGFP966 (2 µM). *N* = 11–12, *p < 0.05 vs. A1^f/f^, ^#^*p* < 0.05 vs. A1^f/f^ LPS. **L** Overall schematic of the study results and mechanism by which A1 dampens the myeloid cell inflammatory response. A1 pathway acts through the downstream enzyme ODC to suppress HDAC3 and IL-1β production. In turn, HDAC3 is involved in TNF-α production and the macrophage pro-inflammatory glycolytic phenotype. HDAC3 suppression by A1/ODC pathway reduces TNF-α and macrophage glycolysis in response to stimulation. The schematic figures were partly generated using Servier Medical Art, provided by Servier, licensed under a Creative Commons Attribution 3.0 unported license”.

We next examined the effect of HDAC3 deletion on macrophage metabolic programming using Seahorse. The HDAC3^-/-^ macrophages exhibited a reduced metabolic switch to glycolysis in response to LPS stimulation as measured by reduced glycolysis and glycolytic capacity (Fig. 6A–C). A Seahorse Mitostress test revealed reduced mitochondrial oxygen consumption rate (OCR) during LPS activation of control HDAC3^f/f^ macrophages, whereas mitochondrial basal and maximal respiration as well as spare respiratory capacity and ATP production were rescued in macrophages of HDAC^-/-^ mice (Fig. S4A–F).

We used the HDAC3 inhibitor, RGFP966 (2 µM), to determine whether HDAC3 mediated the enhanced glycolytic response of LPS-stimulated A1^-/-^ macrophages. Indeed, HDAC3 inhibition decreased glycolysis, glycolytic capacity and glycolytic reserve (as measured by ECAR) in activated A1^-/-^ macrophages (Fig. 6E–H), while basal ECAR was unaffected by RGFP966 in unstimulated macrophages (Fig. 6D). However, RGFP966 did not completely abrogate the increased glycolytic response of LPS-activated A1^-/-^ macrophages suggesting that other mediators may be involved or that pharmacological block of HDAC3 was incomplete. We further conducted a Seahorse ATP Rate Assay (Fig. S4G–I) to measure total ATP production rates and distinguish between the fractions of ATP that are produced from glycolysis and mitochondrial oxidative phosphorylation. In agreement with findings from the glycolysis stress test, LPS-stimulated A1^-/-^ macrophages showed a primary glycolytic phenotype with ~50% of ATP production arising from glycolysis compared to ~25% in stimulated control A1^f/f^ macrophages (Fig. 6I). HDAC3 inhibition with RGFP966 decreased glycolytic ATP production and enhanced mitochondrial ATP production in activated A1^-/-^ macrophages. (Fig. 6J, K). Collectively, these results show that HDAC3 contributes to the glycolytic phenotype observed in activated A1^-/-^ macrophages.

### HDAC3 is expressed in retinal myeloid cells after ischemic injury and further enhanced in A1 KO retinas

**Fig. 7:**
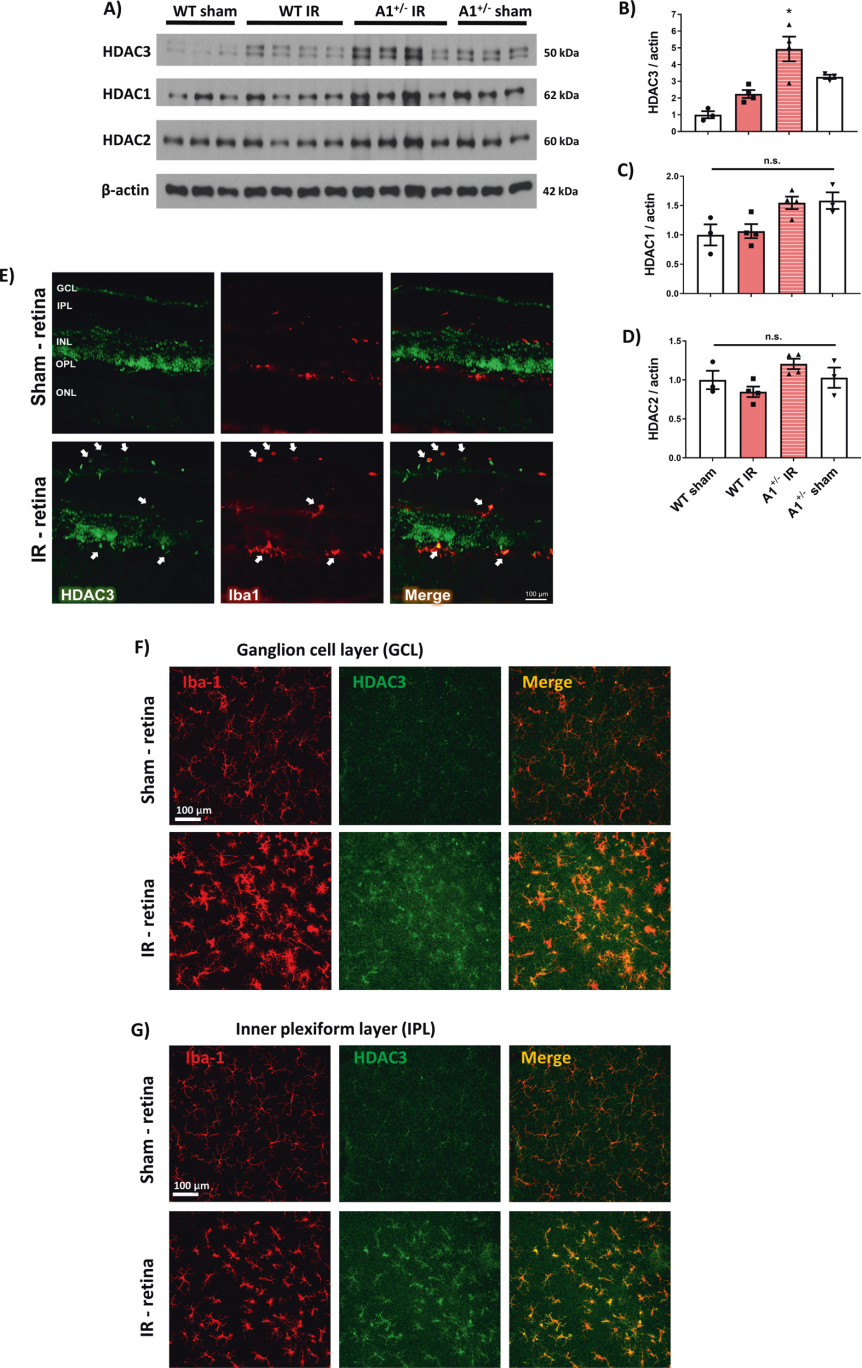
HDAC3 is upregulated in activated myeloid cells in vivo after retinal injury and further increased in A1 KO retinas. **A**–**D** Western blotting and analyses showed upregulation of HDAC3 but not HDAC1 and HDAC2 in the A1^+/-^ retinas at day 2 after IR-injury. *N* = 3–4. *p < 0.05 vs. WT sham and WT IR, n.s. = not statistically significant, two-way ANOVA. **E** HDAC3 immunolabeling studies on retina sections showed baseline expression in the ganglion cell and inner nuclear layers (GCL & INL) while there was a strong co-localization with Iba-1 positive cells after retinal IR-injury. *N* = 3. **F**, **G** HDAC3 immunolabeling on retina flat mounts showed strong expression in Iba-1 positive cells in the ganglion cell and inner plexiform layers (GCL & IPL) after retinal IR-injury. *N* = 3.

Lastly, we examined HDAC3 expression in the injured ischemic retina. Consistent with our findings in LPS-activated A1^-/-^ macrophages, A1^+/-^ retinas showed upregulation of HDAC3, whereas expression levels of HDAC1 and HDAC2 were not significantly increased at day 2 after ischemic injury (Fig. 7A–D). While HDAC3 immunolabeling showed basal neuronal expression in retina sections from sham mice that did not seem to change with injury, co-localization studies and flat mounts showed strong expression of HDAC3 in the Iba-1 positive microglia/macrophages at day 2 after retinal IR injury (Fig. 7E–G). The increase in HDAC3-positive microglia/macrophages extended from the ganglion cell layer to the outer plexiform layer suggesting a critical role of HDAC3 in the retinal inflammatory response after IR.

## Discussion

This study has four principal new findings. First, endogenous arginase 1 (A1) but not A2 limits the induction of IL-1β in retinal macrophages after IR injury in vivo as a possible mechanism of protection; this finding complements our earlier observation that A1 also limits expression of TNF-α in retinal IR injury. Second, the induction by A1 of IL-β1 and TNF-α expression in activated macrophages relies in part on A1 signaling through ODC and formation of polyamines; this A1/ODC/polyamine anti-inflammatory signaling pathway is unique from the recognized A1-mediated reduction in nitric oxide formation that also can exert an anti-inflammatory effect. Third, we show for the first time that a specific histone deacetylase, HDAC3, is involved in the A1/ODC-mediated inhibition of TNF-α but not IL-1β in activated macrophages. Additionally, HDAC3 contributes to the glycolytic response associated with activated macrophages. Fourth, HDAC3 is upregulated in activated microglia/macrophage cells after retinal IR injury and A1 protects against this event. Collectively, our findings indicate that A1 signals through ODC-generated polyamine molecules confer protection from cytokine induction after retinal IR injury partially via HDAC3 inhibition.

Our previous work has documented a protective role of myeloid A1 in retinal IR injury. A1 inhibits the expression of the inflammatory cytokines in activated macrophages in vitro and promotes a retinal neuroprotective phenotype in vivo [2]. The current study characterized the downstream targets that regulate microglia/macrophage activation and cytokine expression after retinal IR injury. We show that microglia/macrophages acquire an ameboid morphology on day 2 days after IR and exhibit a rod-like morphology on day 5. IL-1β is highly upregulated in these microglia/macrophages with a marked increase at day 5. A1 deletion augmented pro-IL-1β expression at day 5 after IR injury, attesting to a protective role of A1 in buffering cytokine production. Similar to IL-1β, TNF-α is upregulated in retinal lysates at day 5 after IR injury, supporting our earlier findings that TNF-α expression is augmented in LPS-stimulated macrophages and acutely in IR-injured retinas when A1 is deleted [2].

The current study provides important new details that argue for polyamines as A1-elicited downstream regulators of IL-1β and TNF-α expression in activated macrophages. Our evidence includes the findings that either A1 deletion or pharmacologic inhibition of polyamine-forming ODC augmented expression of IL-1β and TNF-α in LPS-activated macrophages. A1 deletion also promoted a pro-inflammatory glycolytic phenotype in LPS-stimulated macrophages. Additionally, direct exposure of activated macrophages to ornithine, the primary substrate of ODC, reduced IL-1β expression. Thus, we suggest that the A1/ODC/polyamine signaling pathway represents a significant regulatory mechanism for limiting cytokine production in retinal IR injury. It seems probable that protection offered by A1 signaling through ODC-derived polyamines during retinal IR injury occurs concurrently with its better-recognized protective activity of reducing nitric oxide production by competing with nitric oxide synthase for arginine as a shared substrate. The pleiotropic nature of A1 implies that it likely accesses multiple biochemical pathways to exert its anti-inflammatory effect.

Our study additionally has identified HDAC3 inhibition as a mechanism by which A1-elicited protection from retinal IR injury. As a member of class I HDACs, HDAC3 is essential for the expression of macrophage inflammatory genes mediated by epigenetic modification [19, 20]. Previous studies have shown that HDAC3 negatively regulates A1 expression [19]. In this study, we show that the A1/ODC/polyamine pathway reciprocally and negatively regulates the HDAC3 protein in macrophages. Indeed, inhibition of macrophage A1/ODC/polyamine signaling increased HDAC3 protein expression, whereas conversely increasing availability of A1 using PEG-A1 treatment attenuated HDAC3 levels. Collectively, these data suggest bi-directional negative feedback between the A1/ODC/polyamine pathway and HDAC3. Interestingly, While A1 decreases IL-1β and TNF-α expression in activated macrophages, our results show that HDAC3 is involved in the inhibitory effect of A1 on TNF-α but not IL-1β expression. This could be explained by the presence of specific transcription factors that distinctly regulate IL-1β and TNF-α gene expression [36, 37]. Although the transcription factor NF-κB has been implicated in LPS-mediated IL-1β and TNF-α expression [28], our studies indicate that manipulation of the A1/ODC/polyamine pathway does not affect NF-κB phosphorylation or nuclear translocation.

Macrophages undergo metabolic reprogramming from oxidative phosphorylation to glycolysis under inflammatory conditions [31]. This glycolytic phenotype supports the macrophage proliferative and inflammatory response following activation. Our data show that treatment with PEG-A1 or deletion of HDAC3 dampens this pro-inflammatory glycolytic phenotype. Furthermore, HDAC3 appears to play a key role in the enhanced glycolytic phenotype of LPS-stimulated A1 KO macrophages as measured by the ATP rate assay. Collectively, our results reveal a novel role of HDAC3 suppression in the A1 anti-inflammatory effect in macrophages.

Previously, general HDAC3 inhibitors were found to limit neuronal damage in a rat model of retinal IR injury [38, 39]. One study showed that HDAC3 accounts for 42% of the rat retina total HDAC activity, yet, the study did not detect a change in HDAC3 activity at 24 h after retinal IR injury [40]. In our hands, we detected HDAC3 protein expression in retinal ganglion cells and inner nuclear layers in the control retina with strong expression in Iba-1 positive microglia/macrophages at day 2 after IR. The upregulation of HDAC3 in myeloid cells after retinal IR injury and its further increase in A1 KO retinas suggest a pro-inflammatory epigenetic role of HDAC3 that can hinder the formation of pro-resolving microglia/macrophages.

In conclusion, our collective findings suggest that the anti-inflammatory effect of myeloid-delineated arginase involves A1 signaling through the downstream enzyme ODC to convert ornithine to protective polyamines, which subsequently reduce expression of cytokines IL-β1 and TNF-α in activated macrophages. The A1/ODC-mediated inhibition of TNF-α involves suppression of HDAC3 expression. Based on these findings, HDAC3 in myeloid cells may represent a potential therapeutic target to ameliorate retinal ischemic diseases.

### Supplementary information

Supplementary figure legends

Supplementary figure 1

Supplementary figure 2

Supplementary figure 3

Supplementary figure 4

Uncropped blots

Reproducibility checklist

## Data Availability

Data are available upon request from the authors.
