# Supplementary material for: The arginase 1/ornithine decarboxylase pathway suppresses HDAC3 to ameliorate the myeloid cell inflammatory response: implications for retinal ischemic injury
Source: Cell Death Dis. 2023 Sep 21;14(9):621. doi: 10.1038/s41419-023-06147-7 (PMC10514323; doi:10.1038/s41419-023-06147-7)
Supplement: Supplementary file 1 — Supplementary figure legends [file 41419_2023_6147_MOESM1_ESM.docx]

**Supplementary figure legends:**

**Fig. S1: Iba-1 positive microglia/macrophages exhibit strong proliferation and rod-like morphology at day 5 after IR.**

Flatmount staining for Iba-1 and 3D image reconstruction shows a ramified microglia morphology in the sham retina. The microglia acquired a more ameboid morphology with enlarged soma and retracted processes at day 2 after IR injury. The Iba-1 positive microglia/macrophages increased in number and acquired a rod-like structure at day 5 after IR injury. N=5.

**Fig S2: A1 does not affect NF-κB nuclear translocation in response to LPS stimulation.**

(A) Quantification of A1 band from the Western blot in figure 2A showing no change in A1 protein levels with A2 deletion or LPS and PEG-A1 treatment. N=4, n.s. = not statistically significant.

(B, C) Western blotting showed no change in A1 protein levels with DFMO (5 mM) treatment in LPS stimulated macrophages. N=3, n.s. = not statistically significant.

(D) Confirmation of A1 deletion in A1^-/-^ macrophages with Western blotting showing no band for A1. N=3.

(E, F) PEG-A1 (1 µg/ml) treatment did not affect LPS-induced NF-κB nuclear translocation as measured by immunocytochemistry. Scale bar = 25 μm, N=5, *p<0.05 vs respective control.

(G, H) NF-κB nuclear translocation was not different between control and A1 KO macrophages. N=4-5, Scale bar = 25 μm, *p<0.05 vs respective control.

**Fig S3: A1 deletion does not affect ODC or HDAC10 expression.**

(A, B) RT-PCR shows no change in the LPS-induced increases in mRNA levels of ODC or the polyamine deacetylase HDAC10. N=6, *p<0.05 vs respective control.

(C) PEG-A1 treatment reduces HDAC3 protein expression in A1 KO macrophages stimulated with LPS.

(D, E) A1 protein expression is increased in HDAC3 KO macrophages. N=6. *p<0.05 vs HDAC3^f/f^.

**Fig S4: HDAC3 deletion ameliorates the LPS-induced inhibition of mitochondrial respiration in macrophages.**

(A-F) Unstimulated HDAC3 KO BMDMs showed increased basal respiration and ATP production while maximal respiration, spare respiratory capacity and coupling efficiency decreased. Upon LPS stimulation, HDAC3 KO macrophages showed higher mitochondrial respiration parameters relative to the LPS treated floxed macrophages. *p<0.05 vs HDAC3^f/f^, ^$^p<0.05 vs HDAC3^f/f^ and HDAC3^f/f^ LPS, ^#^p<0.05 vs HDAC3^f/f^ LPS.

(G-I) OCR, ECAR and PER graphs for the ATP rate assay.
