# Supplementary figures and images for: The arginase 1/ornithine decarboxylase pathway suppresses HDAC3 to ameliorate the myeloid cell inflammatory response: implications for retinal ischemic injury
Source: Cell Death Dis. 2023 Sep 21;14(9):621. doi: 10.1038/s41419-023-06147-7 (PMC10514323; doi:10.1038/s41419-023-06147-7)

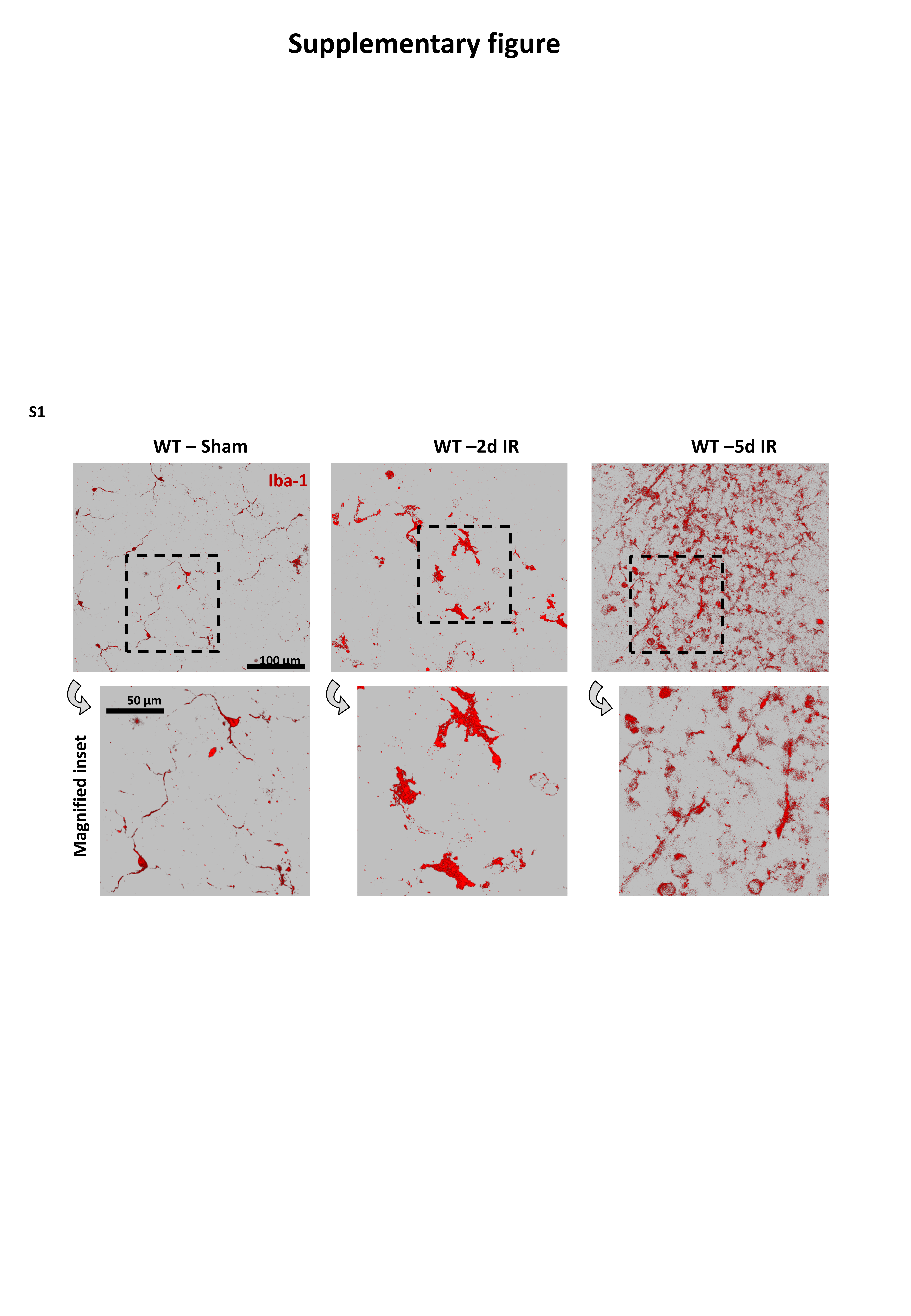

Supplement: Supplementary file 2 — Supplementary figure 1 [file 41419_2023_6147_MOESM2_ESM.tif]

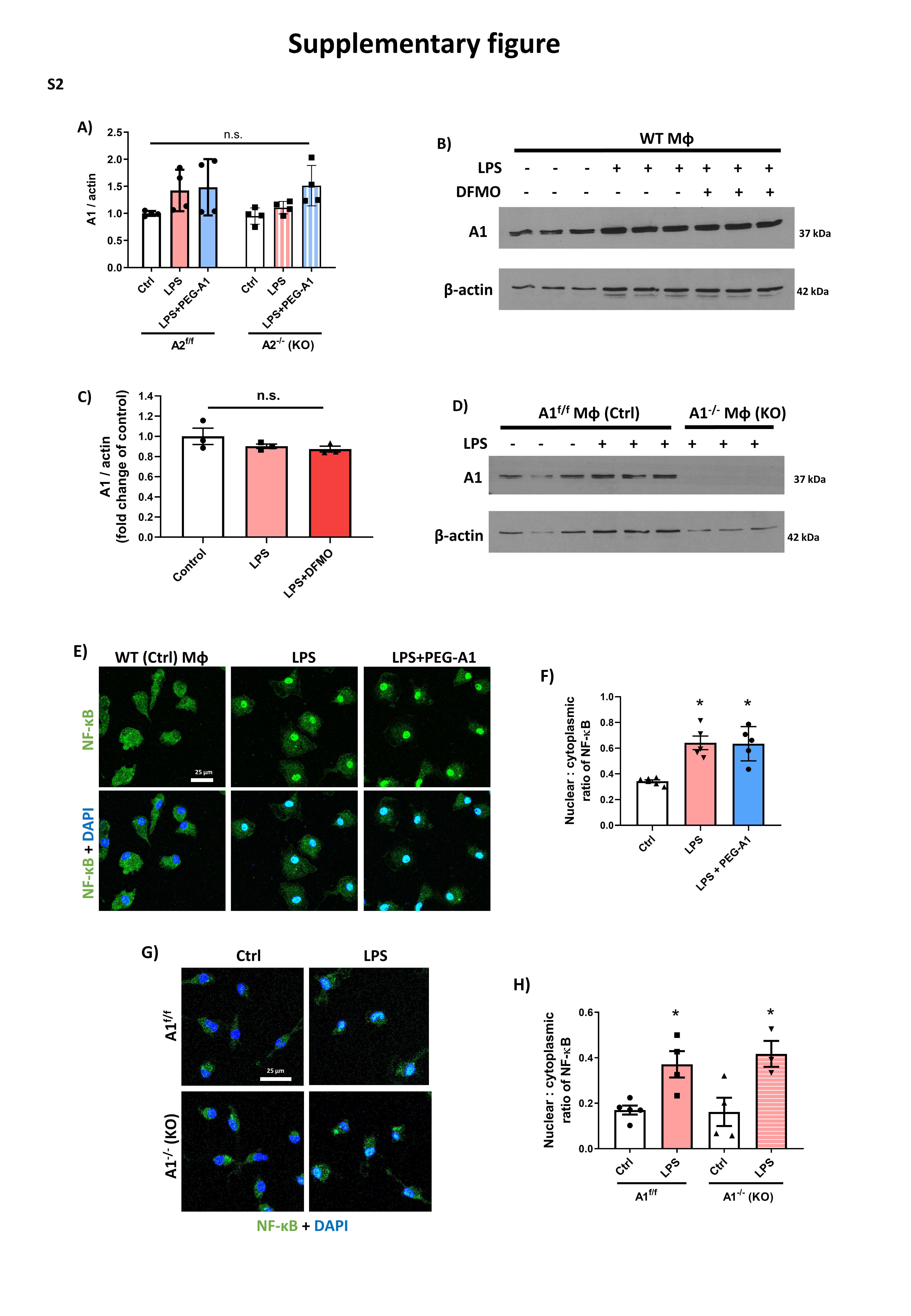

Supplement: Supplementary file 3 — Supplementary figure 2 [file 41419_2023_6147_MOESM3_ESM.tif]

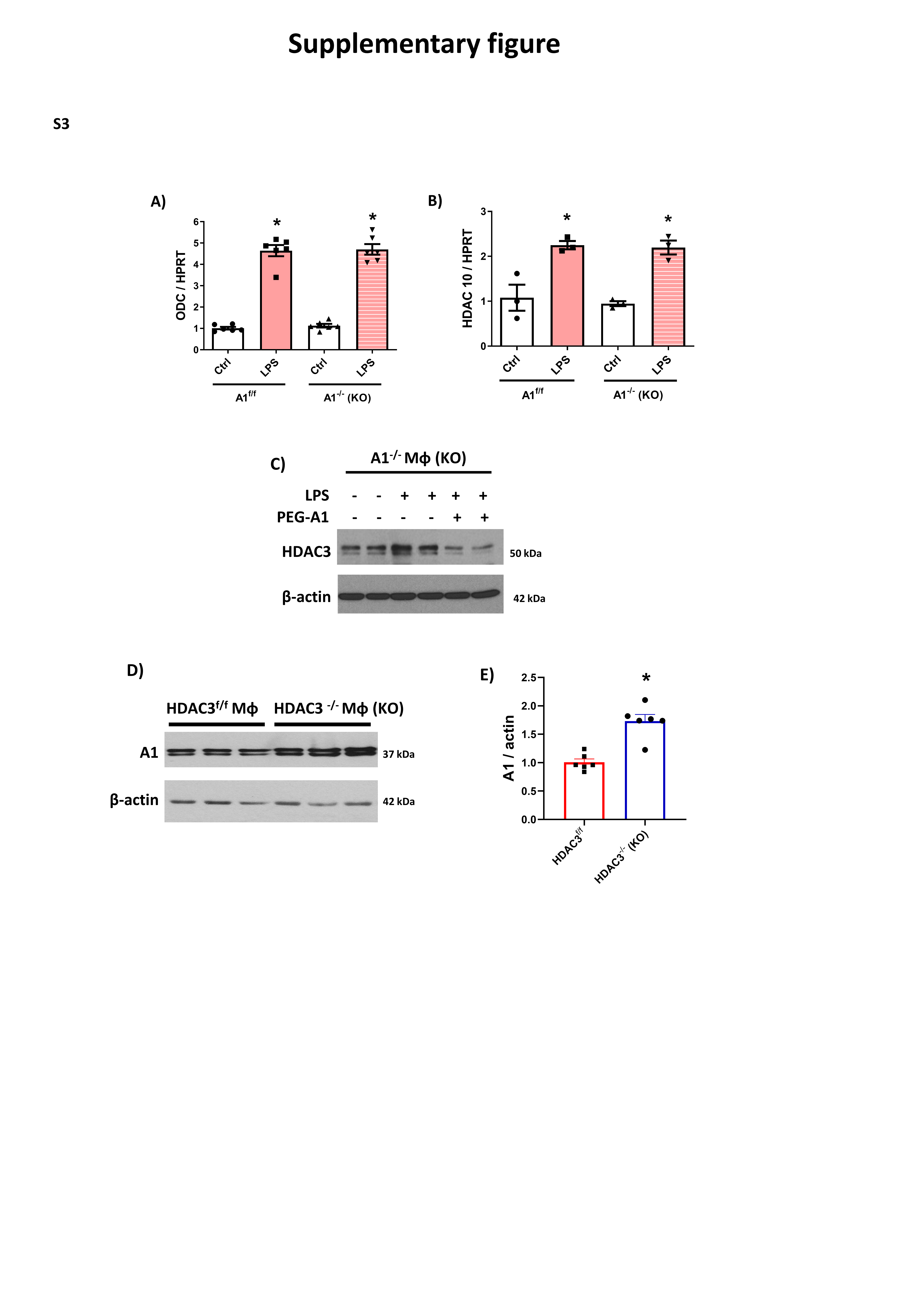

Supplement: Supplementary file 4 — Supplementary figure 3 [file 41419_2023_6147_MOESM4_ESM.tif]

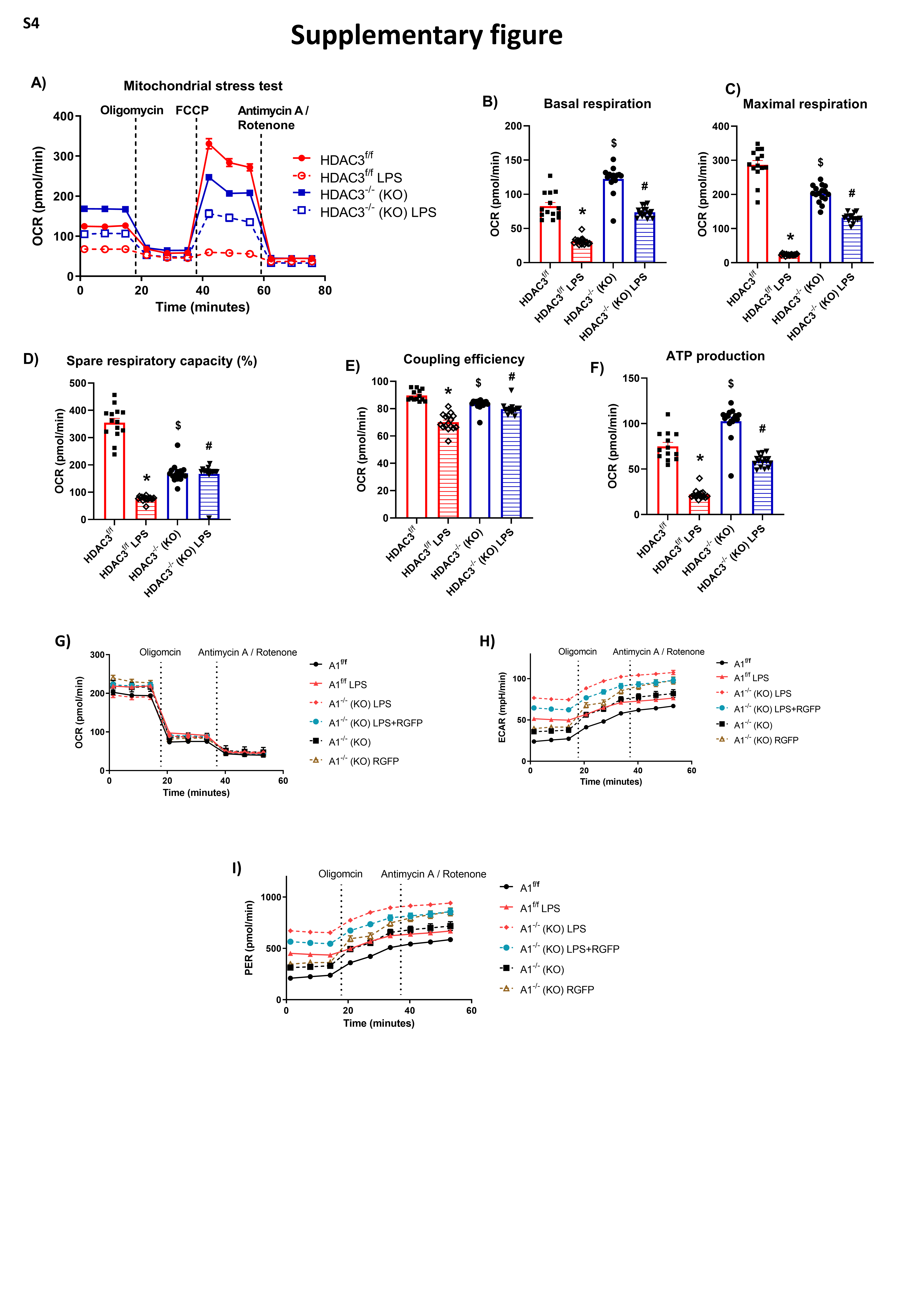

Supplement: Supplementary file 5 — Supplementary figure 4 [file 41419_2023_6147_MOESM5_ESM.tif]
