# Supplementary material for: The arginase 1/ornithine decarboxylase pathway suppresses HDAC3 to ameliorate the myeloid cell inflammatory response: implications for retinal ischemic injury
Source: Cell Death Dis. 2023 Sep 21;14(9):621. doi: 10.1038/s41419-023-06147-7 (PMC10514323; doi:10.1038/s41419-023-06147-7)

Uncropped Western blots

F1

A)

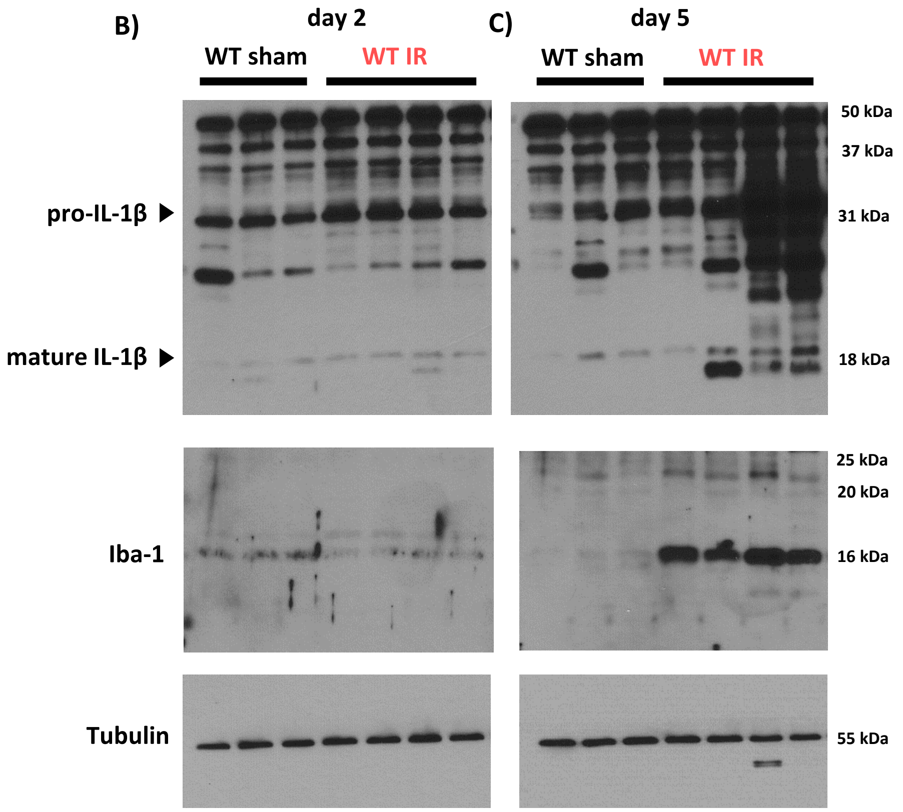
B)
day 2
WT sham
WT IR
C)
day 5
WT sham
WT IR
pro-IL-1β
mature IL-1β
Iba-1
Tubulin
50 kDa
37 kDa
31 kDa
18 kDa
25 kDa
20 kDa
16 kDa
55 kDa

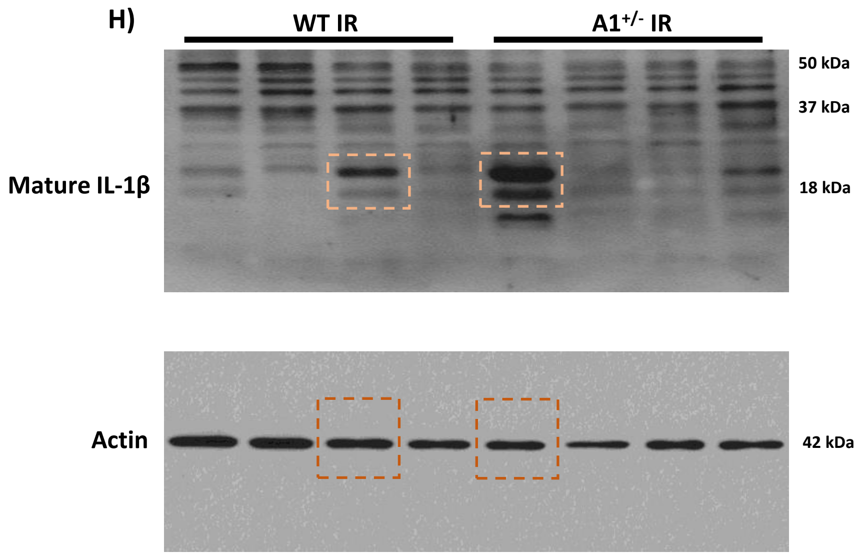
H)
WT IR
A1+/- IR
Mature IL-1β
Actin
50 kDa
37 kDa
18 kDa
42 kDa

F2

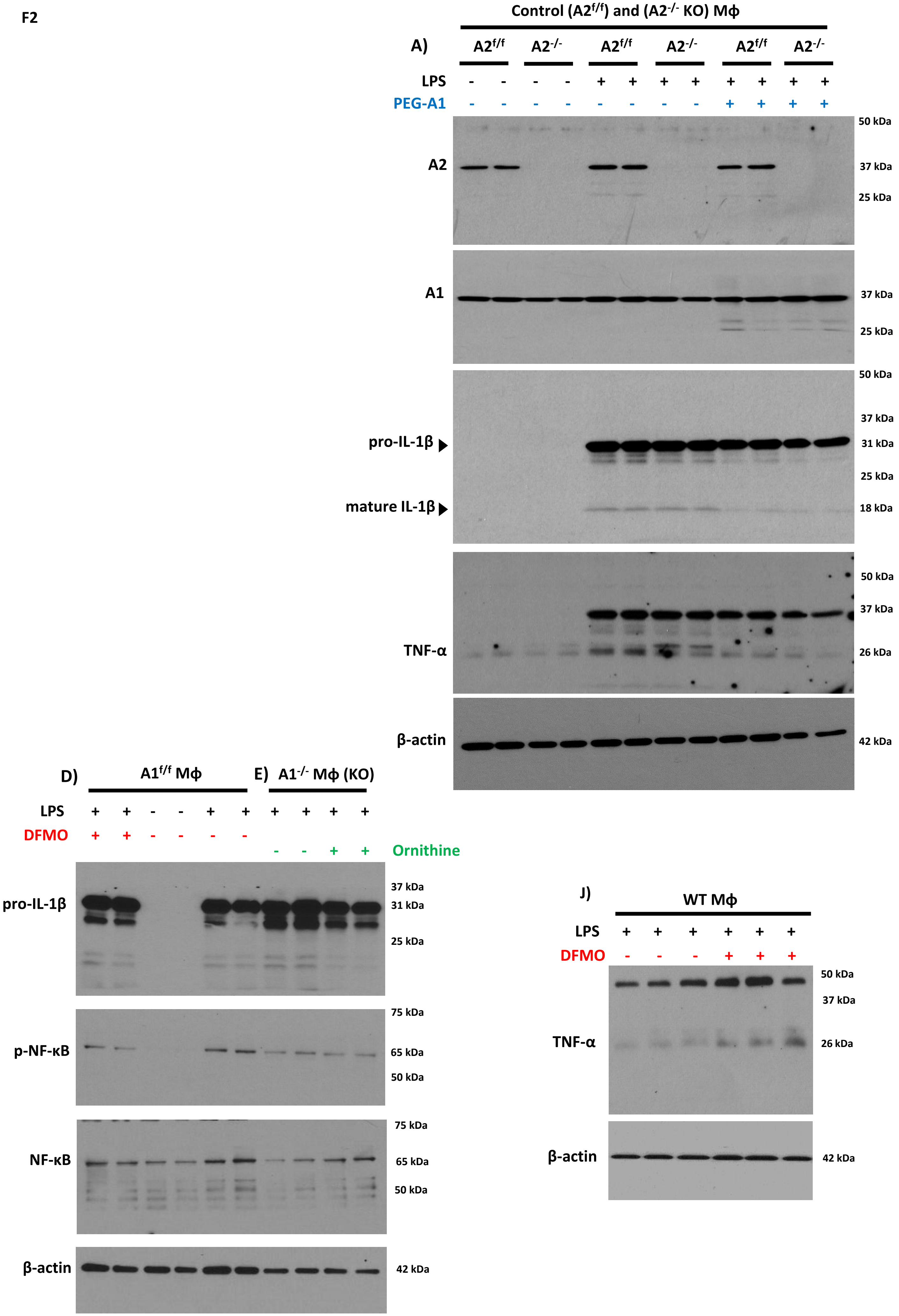

Control (A2f/f) and (A2-/- KO) Mϕ
A)
A2f/f
A2-/-
A2f/f
A2-/-
A2f/f
A2-/-
LPS
PEG-A1
A2
A1
pro-IL-1β
mature IL-1β
TNF-α
β-actin
50 kDa
37 kDa
25 kDa
37 kDa
25 kDa
50 kDa
37 kDa
31 kDa
25 kDa
18 kDa
50 kDa
37 kDa
26 kDa
42 kDa
D)
A1f/f Mϕ
E)
A1-/- Mϕ (KO)
LPS
DFMO
Ornithine
pro-IL-1β
p-NF-κB
NF-κB
β-actin
37 kDa
31 kDa
25 kDa
75 kDa
65 kDa
50 kDa
75 kDa
65 kDa
50 kDa
42 kDa
J)
WT Mϕ
LPS
DFMO
TNF-α
β-actin
50 kDa
37 kDa
26 kDa
42 kDa

F3

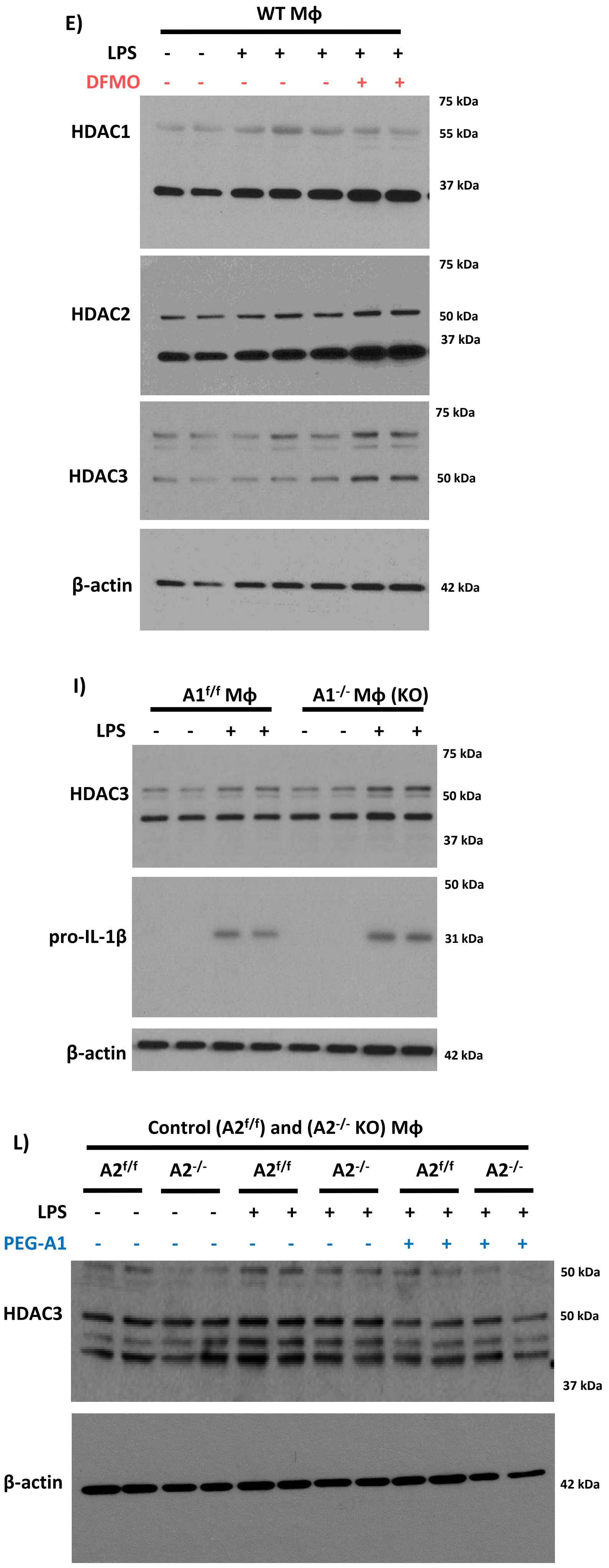
E)
WT Mϕ
LPS - - + + + + +
DFMO - - - - - + +
HDAC1
75 kDa
55 kDa
37 kDa
HDAC2
75 kDa
50 kDa
37 kDa
HDAC3
75 kDa
50 kDa
β-actin
42 kDa
I)
A1$^{f/f}$ Mϕ
A1$^{-/-}$ Mϕ (KO)
LPS - - + + - - + +
HDAC3
75 kDa
50 kDa
37 kDa
pro-IL-1β
50 kDa
31 kDa
β-actin
42 kDa
L)
Control (A2$^{f/f}$) and (A2$^{-/-}$ KO) Mϕ
A2$^{f/f}$
A2$^{-/-}$
A2$^{f/f}$
A2$^{-/-}$
A2$^{f/f}$
A2$^{-/-}$
LPS - - - - + + + + + + + +
PEG-A1 - - - - - - - - + + + +
HDAC3
50 kDa
50 kDa
37 kDa
β-actin
42 kDa

F4

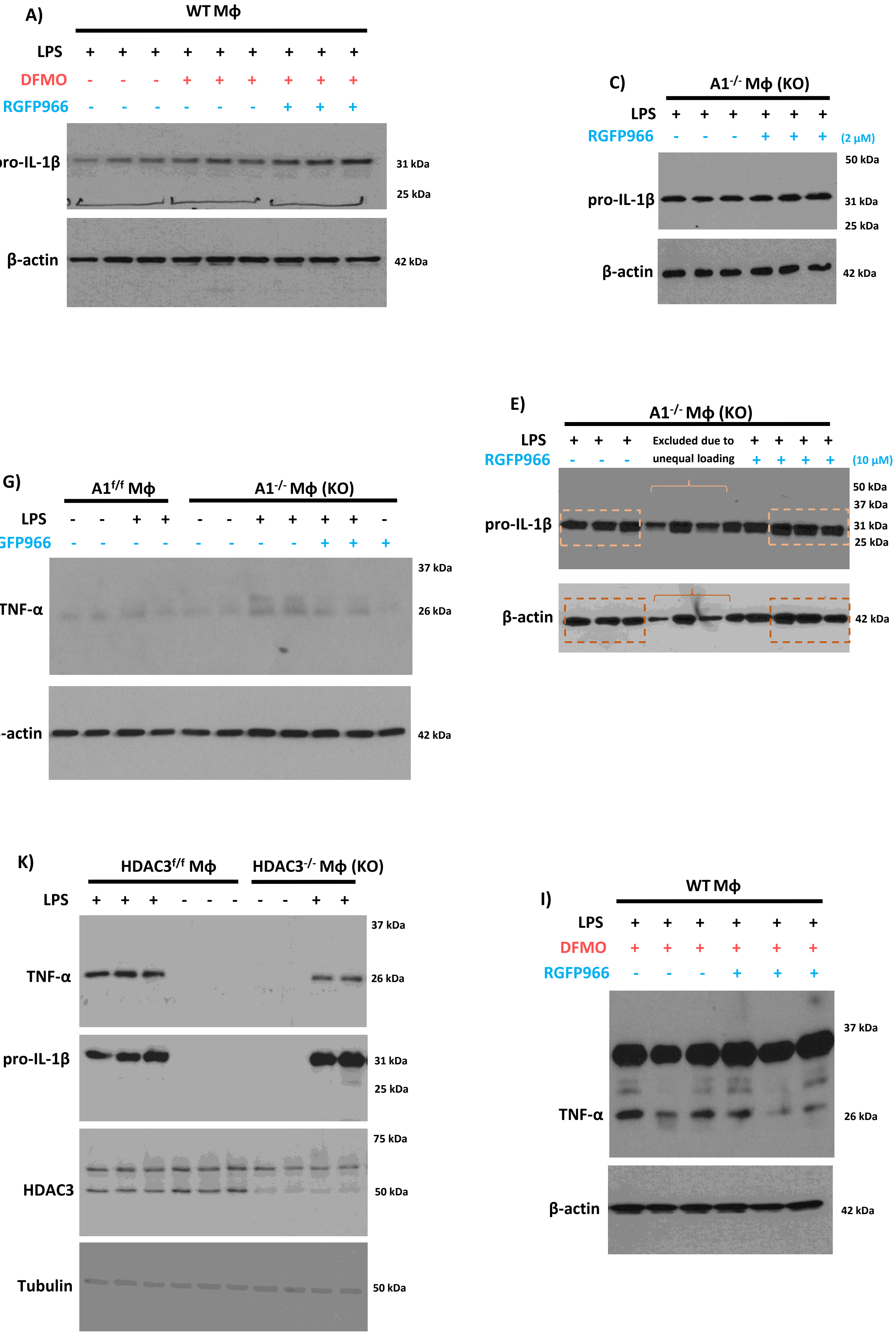
A)
WT Mϕ
LPS
DFMO
RGFP966
pro-IL-1β
31 kDa
25 kDa
β-actin
42 kDa
C)
A1-/- Mϕ (KO)
LPS
RGFP966
(2 μM)
50 kDa
pro-IL-1β
31 kDa
25 kDa
β-actin
42 kDa
E)
A1-/- Mϕ (KO)
LPS
RGFP966
Excluded due to
unequal loading
(10 μM)
50 kDa
37 kDa
pro-IL-1β
31 kDa
25 kDa
β-actin
42 kDa
G)
A1f/f Mϕ
A1-/- Mϕ (KO)
LPS
RGFP966
37 kDa
TNF-α
26 kDa
β-actin
42 kDa
K)
HDAC3f/f Mϕ
HDAC3-/- Mϕ (KO)
LPS
37 kDa
TNF-α
26 kDa
pro-IL-1β
31 kDa
25 kDa
75 kDa
HDAC3
50 kDa
Tubulin
50 kDa
I)
WT Mϕ
LPS
DFMO
RGFP966
37 kDa
TNF-α
26 kDa
β-actin
42 kDa

F7

A)

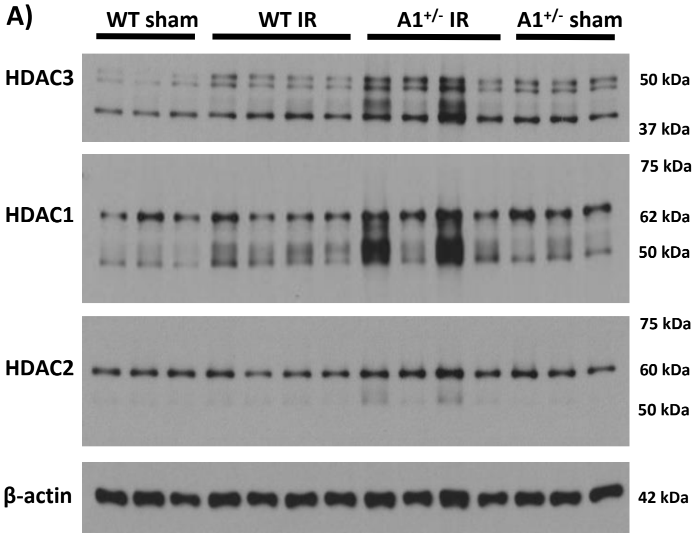

Supplementary figures

S2

A)

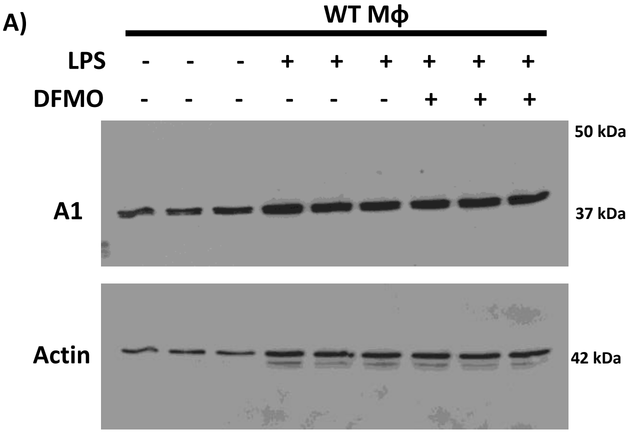

C)

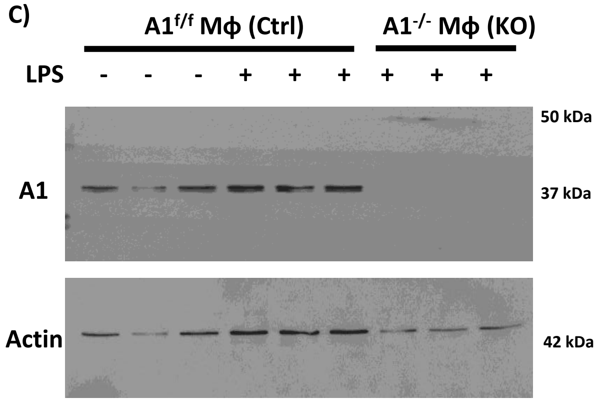

S3

C)

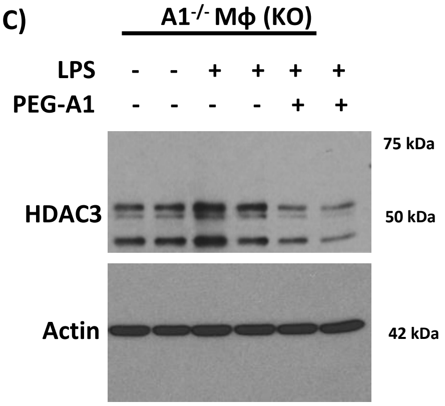

D)

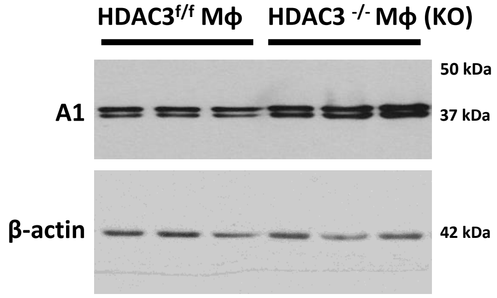

Supplement: Supplementary file 6 — Uncropped blots [file 41419_2023_6147_MOESM6_ESM.pdf]
